# Paeoniflorin protects hepatocytes from APAP-induced damage through launching autophagy via the MAPK/mTOR signaling pathway

**DOI:** 10.1186/s11658-024-00631-4

**Published:** 2024-09-07

**Authors:** Xinyu Deng, Yubing Li, Yuan Chen, Qichao Hu, Wenwen Zhang, Lisheng Chen, Xiaohua Lu, Jinhao Zeng, Xiao Ma, Thomas Efferth

**Affiliations:** 1 State Key Laboratory of Southwestern Chinese Medicine Resources, School of Pharmacy, Chengdu University of Traditional Chinese Medicine, Chengdu, 611137 China; 2 TCM Regulating Metabolic Diseases Key Laboratory of Sichuan Province, Hospital of Chengdu University of Traditional Chinese Medicine, Chengdu, 610072 China; 3 Department of Pharmaceutical Biology, Institute of Pharmaceutical and Biomedical Sciences, Johannes Gutenberg University, Mainz, 55128 Germany

**Keywords:** Acetaminophen, Cell death, Drug-induced liver injury, Natural products, Oxidative stress, Signal transduction

## Abstract

**Background:**

Drug-induced liver injury (DILI) is gradually becoming a common global problem that causes acute liver failure, especially in acute hepatic damage caused by acetaminophen (APAP). Paeoniflorin (PF) has a wide range of therapeutic effects to alleviate a variety of hepatic diseases. However, the relationship between them is still poorly investigated in current studies.

**Purpose:**

This work aimed to explore the protective effects of PF on APAP-induced hepatic damage and researched the potential molecular mechanisms.

**Methods:**

C57BL/6J male mice were injected with APAP to establish DILI model and were given PF for five consecutive days for treatment. Aiming to clarify the pharmacological effects, the molecular mechanisms of PF in APAP-induced DILI was elucidated by high-throughput and other techniques.

**Results:**

The results demonstrated that serum levels of ALP, γ-GT, AST, TBIL, and ALT were decreased in APAP mice by the preventive effects of PF. Moreover, PF notably alleviated hepatic tissue inflammation and edema. Meanwhile, the results of TUNEL staining and related apoptotic factors coincided with the results of transcriptomics, suggesting that PF inhibited hepatocyte apoptosis by regulated MAPK signaling. Besides, PF also acted on reactive oxygen species (ROS) to regulate the oxidative stress for recovery the damaged mitochondria. More importantly, transmission electron microscopy showed the generation of autophagosomes after PF treatment, and PF was also downregulated mTOR and upregulated the expression of autophagy markers such as *ATG5*, *ATG7*, and *BECN1* at the mRNA level and LC3, p62, ATG5, and ATG7 at the protein level, implying that the process by which PF exerted its effects was accompanied by the occurrence of autophagy. In addition, combinined with molecular dynamics simulations and western blotting of MAPK, the results suggested p38 as a direct target for PF on APAP. Specifically, PF-activated autophagy through the downregulation of MAPK/mTOR signaling, which in turn reduced APAP injury.

**Conclusions:**

Paeoniflorin mitigated liver injury by activating autophagy to suppress oxidative stress and apoptosis via the MAPK/mTOR signaling pathway. Taken together, our findings elucidate the role and mechanism of paeoniflorin in DILI, which is expected to provide a new therapeutic strategy for the development of paeoniflorin.

**Graphical abstract:**

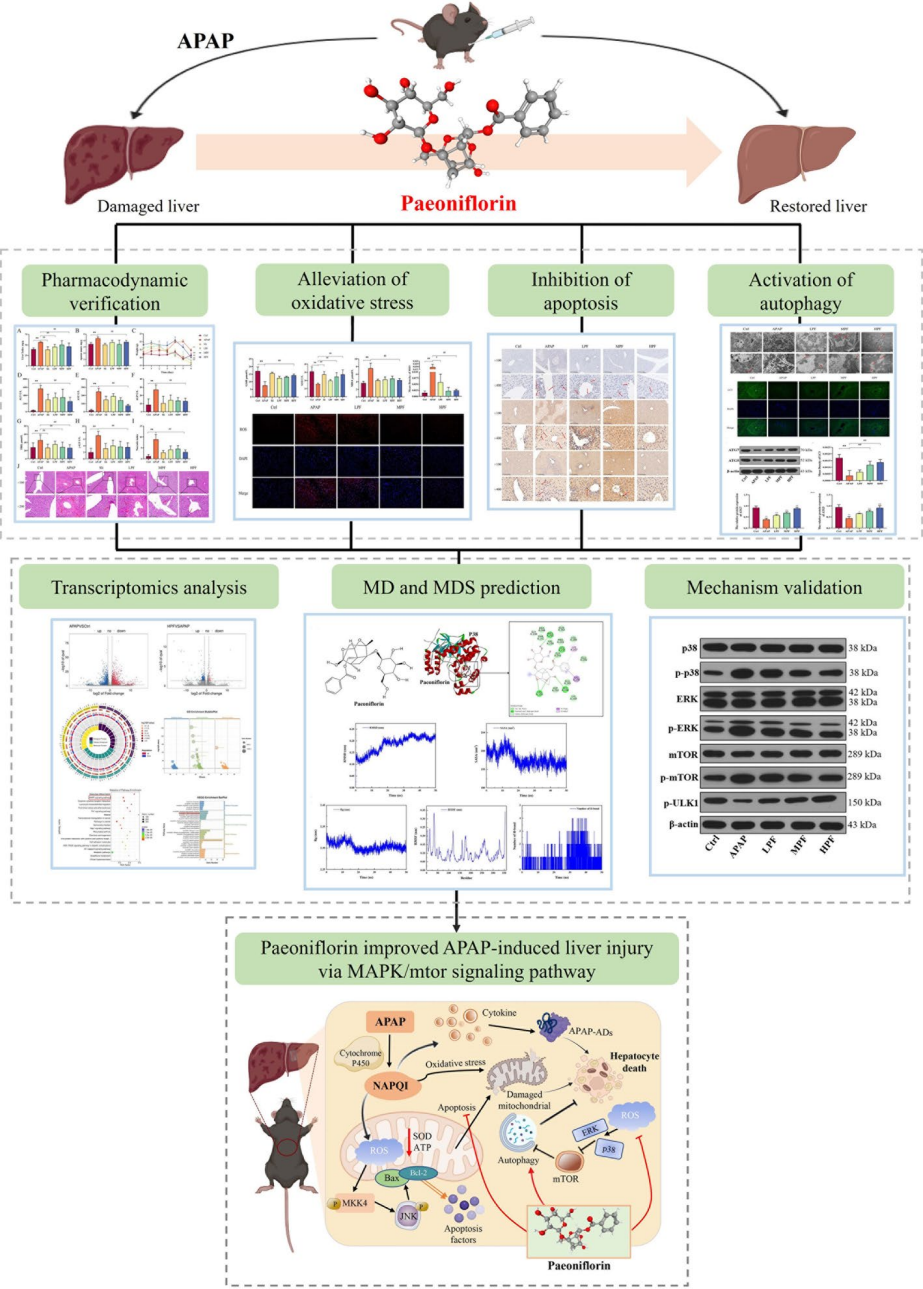

**Supplementary Information:**

The online version contains supplementary material available at 10.1186/s11658-024-00631-4.

## Introduction

Drug-induced liver injury (DILI) refers to liver damage caused by drugs or their metabolites and is one of the most common forms of adverse drug reactions. Many drugs, especially nonsteroidal anti-inflammatory drugs (NSAIDs), anticancer drugs and antibiotics, can cause DILI to varying degrees in clinical use [1–3]. One commonly used NSAID, acetaminophen (APAP, Figure S1A), is a leading cause of pharmacological acute liver failure [4]. APAP-induced DILI is mainly caused by its toxic metabolite, *N*-acetyl-*p*-benzoquinone imine (NAPQI), which has a complex mechanism of toxicity, including interference with hepatic metabolism, induction of mitochondrial oxidative stress and dysfunction, promotion of hepatocyte necrosis, activation of inflammatory responses, endoplasmic reticulum stress, and autophagic processes [5, 6]. Autophagy, as an intracellular protective mechanism, contributes to the removal of damaged organelles and protein aggregates, thereby reducing the metabolic burden and oxidative stress of hepatocytes and protecting them from further damage [7]. The current study suggested that inhibition of autophagy might be an early cause of drug-induced liver injury, while promotion of autophagy helped to attenuate its occurrence [8, 9]. In the APAP-induced DILI model, researchers found that proper regulation of autophagy could effectively remove APAP protein adducts (APAP-ADs) from hepatocytes, ameliorate mitochondrial dysfunction and necrosis, and promote hepatocyte repair and regeneration, thereby effectively reducing the severity of liver injury [10]. Therefore, modulation of the autophagy process may become a potential strategy for the treatment of drug-induced liver injury.

The mitogen-activated protein kinase (MAPK) and mammalian target of rapamycin (mTOR) signaling pathways together form a complex and critical signaling network in the cell. These pathways involve a variety of key protein kinases and regulators, including the MAPK family such as extracellular signal-regulated kinase (ERK), c-Jun N-terminal kinase (JNK), p38 MAPK, and mTOR [11]. The MAPK family regulates cell growth, proliferation, inflammatory response, and apoptosis through phosphorylation, whereas mTOR is a key regulatory protein mainly involved in the regulation of cellular metabolism, growth, and autophagy pathways [12]. In particular, when regulated by upstream signals, mTOR is able to influence the onset of autophagy, modulate intracellular autophagic flux, and contribute to the clearance of damaged organelles and toxic metabolites [13]. In the context of pharmacological liver injury, changes in mTOR expression are critical for the regulation of autophagy. Abnormal activation of mTOR often leads to inhibition of autophagy, thereby exacerbating hepatocyte injury [14, 15]. Meanwhile, activation or inhibition of upstream signals in the MAPK/mTOR pathway, in particular alterations in kinases such as ERK, JNK, and P38, have been shown to play a key role in liver injury [16]. Therefore, modulation of these pathways, particularly targeting MAPK signaling, may be one of the potential strategies for the treatment of drug-induced liver injury.

Natural products are bioactive substances extracted from plants or minerals, and in recent years there have been many reports confirming the potential therapeutic activity of natural products in the treatment of DILI by regulating MAPK signaling [17, 18]. Paeoniflorin (PF, Figure S1B) is a monoterpene glycoside extracted from the Chinese herbs *Paeonia suffruticosa* Andr., *Paeonia lactiflora* Pall., or *Paeonia veitchii* Lynch. and has a wide range of pharmacological activities, including anti-apoptotic, antioxidant, and anticancer properties, with potential therapeutic effects on a wide range of diseases [19, 20]. In particular, paeoniflorin has significant hepatoprotective effects and can be used for the treatment of liver fibrosis, cholestasis, nonalcoholic fatty liver disease, and hepatocellular carcinoma [21]. In our previous study, paeoniflorin provided initial protection against APAP-induced liver injury by inhibiting JNK-related signaling, thus validating the potential of paeoniflorin for the treatment of APAP-induced DILI [22]. To provide new perspectives on the pharmacological activity of paeoniflorin, we further elucidated the molecular mechanism of paeoniflorin in the treatment of APAP-induced DILI in the present study.

## Materials and methods

### Chemicals and reagents

The details of acetaminophen, paeoniflorin, and silibinin (Sli, positive drug) are shown in Table S1. Prior to use, PF and Sli were prepared in saline. APAP was dissolved in hot saline at a temperature of 66 ℃ in advance for use. Biochemical indicators and oxidative stress-related indicators for measure were included, namely ALT, ALP, γ-GT, TBIL, AST, GSH, SOD, and MDA (Table S1). Other chemicals not otherwise specified were of analytical grade and had been received from commercial sources.

### Experimental animals

The study procedures and animal care have been approved by the animal ethics committee (no. TCM-2022-305). Adult C57BL/6J mice (aged 7–8 weeks, male, averaged 20–24 g) were acquired from SiPaiFu Biotechnology Co. (Beijing, China) (license no.: SCXK (Jing) 2019-0010). Healthy mice were acclimatized and fed for one week under standard conditions (50% relative humidity; room temperature of 22–25 ℃ accompanied by 12 h of alternating light and darkness; free diet and water) in the Specific pathogen Free (SPF) standard environment at the Animal Experimentation Center of Chengdu University of Traditional Chinese Medicine (SYXK (Chuan) 2020–124) before the experiment.

### DILI model and treatment

The experimental animals were randomly allocated into six groups in a blinded manner before receiving the administration, including the control group (ctrl), model group (APAP), silibinin group (Sli), low-dose paeoniflorin group (LPF), middle-dose paeoniflorin group (MPF), and high-dose paeoniflorin group (HPF) (Table S2). The therapeutic drugs were given by gavage for five consecutive days in all groups except control and APAP groups, which were given saline. After the last administration, fasting was performed for 12 h. DILI model was established by intraperitoneal injection of APAP, meanwhile, equal amount of saline was injected in control group. A total of 12 h after model establishment, the mice were killed with an appropriate amount of urethane. Sera and hepatic tissues were collected immediately after sacrifice, rinsed in ice saline, and stored or processed as required for the sorts of experiments such as pathological staining, western blotting, etc.

### Biochemical analysis

Hepatic function indicators and oxidative stress-related indicators in serum were measured by colorimetric determination, according to the manufacturer’s specification (Nanjing Jiancheng Bioengineering Institute, Nanjing, China).

### Histological examination by hematoxylin–eosin (H&E) staining

Dehydration, fixation, paraffin embedding, and H&E staining were performed on hepatic samples preserved in 4% paraformaldehyde. Light microscopy was used to observe the pathology and the score of necrosis was indicated by the necrotic index, which represented the number of focal necrosis upon low magnification (×100, at least ten fields of view) in each group of samples was determined blindly [23].

### Transcriptomic analysis

According to the manufacturer’s instructions, total RNA was extracted from frozen hepatic tissues using Trizol reagent (Thermo Fisher, 15596018). The quantity and purity of total RNA were analyzed by RNA 6000 Nano LabChip Kit (Agilent, CA, USA, 5067-1511) and Bioanalyzer 2100. Among them, eligible RNA sequences were selected for library construction. After two purifications, the mRNA was fragmented into short fragments under the guidance of kit (Magnesium RNA Fragmentation Module) at high temperature (94 ℃, 5–7 min). After reverse transcription to cDNA, following the instructions so that the average insert fragment in the final complementary DNA libraries were 300 ± 50 bp. Finally, we carried out the 2 × 150 bp paired-end sequencing (PE150) on a Novaseq™ 6000 (Illumina, San Diego, America), following the protocol recommended by the manufacturer. The follow-up sequencing experiments were entrusted to Shanghai BIOTREE Biomedical Technology Co., Ltd. (Shanghai, China) and the final data obtained were subjected to bioinformatics analysis, such as GO analysis (http://geneontology.org) and KEGG analysis (https://www.kegg.jp/kegg/).

### Molecular docking and molecular dynamics simulations

Molecular docking was used to model the binding and interaction of PF with the core target of APAP hepatotoxicity. On the one hand, the structure of PF was downloaded from PubChem with CID 442534. On the other hand, the core protein information was searched and downloaded as PDB format files through the protein database (http://www.rcsb.org/) [24]. The PDBQT protein receptor file was obtained from AutoDock4 by removing the water and replacing it with hydrogen. The same operation was carried out for small molecules and simulations were performed with protein as receptors and compounds as ligands to gain the fitted minimum binding energy. At last, the composite files in Protein Data Bank (PDB) format were imported by PyMol2.5 (https://pymol.org/2/) for visualization. GROMACS software (2020.3, https://www.gromacs.org/) was used to exert the molecular dynamics simulations. The parameter and topology of proteins and ligands were respectively generated by the general Amber force field (GAFF) and the amber99sb-ildn force. The selection of 1.4 nm was used to judge the nonbond truncation distance and the Van der Waals forces were calculated by Lennard–Jones function. The bond length of all atoms was constrained by the LINCS algorithm [25]. The long-range electrostatic interaction was calculated by the Particle Mesh-Ewald method with the Fourier spacing 0.16 nm [26].

### Immunohistochemistry (IHC)

The 4% paraformaldehyde-fixed samples were paraffin-embedded, and the embedded sections were dewaxed, washed, and repaired with citric acid on high fire. Sequentially, 3% H_2_O_2_ and BSA were added for closure. The sections were incubated with primary antibody overnight at 4 °C. After washing three times with PBS, the secondary antibody was incubated for 50 min and stained with diaminobenzidine (DAB) at room temperature. All antibodies used are displayed in Table S3. We used ImageJ software (version 1.54i) to analyze the positive expression of antigen [22].

### Immunofluorescence (IF)

The 4% paraformaldehyde-fixed hepatic tissues were stored at room temperature and the subsequent experimental procedure was similar to that of IHC experiments except that fluorescent secondary antibodies were used for incubation. DAPI staining solution was used to re-stain cell nucleus. Positive areas were observed under fluorescence microscope (Eclipse Ti-SR, Nikon, Japan) and counted by ImageJ (version 1.54i).

### TUNEL assay for apoptosis

Following the instructions of the TUNEL peroxidase apoptosis detection kit (cat.: 11684817910, Roche, Shanghai, China), the embedded completed hepatic sections were de-stained and rehydrated. The sections were stained and reacted with fluorescein labeling solution in a moist dark environment. After rinsing three times with PBS, DAB was added as substrate and reacted for 10 min and washed again. The processed materials were placed under a light microscope for identification.

### Transmission electron microscopy (TEM) experiments

The hepatic tissue samples were fixed in 5% glutaraldehyde for at least 5 h at 4 ℃, followed by three washes with phosphate buffer and neutral buffer every 10 min. Subsequently, the tissues were fixed in 0.1 mol/L osmium acid for 3 h and accompanied by phosphate buffer washing every 10 min. This was followed by gradient dehydration using ethanol in different concentrations every 15 min. The tissues were then subjected to resin infiltration, embedding, polymerization, and finally made into filminess sections (60–80 nm). These sections were dual stained with uranyl acetate and lead citrate, air-dried at room temperature, and photographed under a transmission electron microscope (JEM-1400FLASH, JEOL, Japan).

### RNA isolation and real-time quantitative polymerase chain reaction (PCR)

Total RNA was isolation from liver using Foregene kit (no. RE-03011/03014, Chengdu, China) according to the guideline from the manufacturer. The extracted total RNA was reverse transcribed to cDNA in the order of cleaning, purification, and reverse transcription. Samples were mixed with the target gene primers and co-reacted, and at least three technical replicates were followed for each sample to ensure homogeneity. The PCR experiment was terminated by analyzing the melting curve to guarantee the specificity of the expected products of PCR. The primer sequences were designed by ourselves, and the sequence synthesis was performed by Beijing Kengke Biotechnology Co, Ltd. (Beijing, China), on the basis of mRNA sequence received from the NCBI (https://www.ncbi.nlm.nih.gov/). Full details of mRNA sequences are presented in Table S4. At last, the levels of target mRNAs were standardized to *GAPDH* mRNA. Then, we utilized the ΔΔ^−Ct^ method to calculate the qPCR data.

### Protein extraction and western blot (WB) analysis

Proteins were extracted from the hepatic samples as the previously procedures [22]. Further, through the process of electrophoretic separation, membrane transfer, containment, cleaning, incubation of primary antibody (12–15 h), and secondary antibody incubation (60 min), the complete bands were finally developed. β-Actin was regarded as the control in the experiments, and the results of protein expression were gained by adjusting the grayness and background of bands. The information of antibodies is given in Table S3.

### Data and statistical analysis

Total data were analyzed by SPSS program (version 25.0) and embodied as mean ± standard deviation (SD) from at least three replicates. The results satisfied the Gaussian distribution and could be adopted by the one-way analysis of variance (ANOVA) analysis; conversely, non-parametric tests were performed. The *p* < 0.05 was labeled as the significant values between groups. ImageJ (version 1.54i) was embodied to analyze the positive expression of the experiments such as IHC and IF. Beyond that, the visualization of the results was presented by Graph Pad Prism (version 8.0, Boston, America).

## Results

### PF attenuates the pathological changes in liver injury generated by APAP

**Fig. 1:**
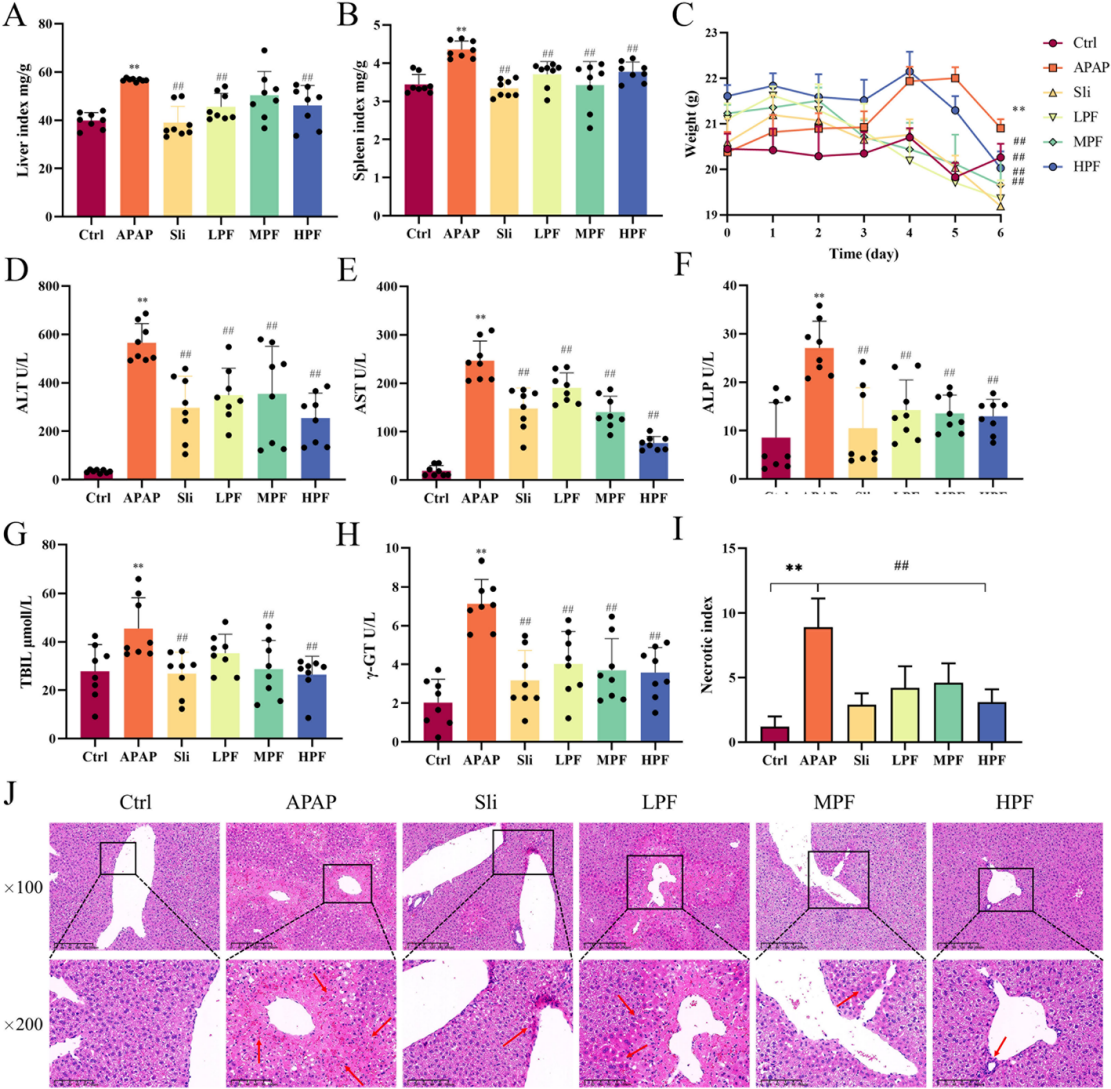
Biochemical and pathological parameters changes by PF on APAP-induced mice. The levels of liver index (**A**) and spleen index (**B**) are shown (*n* = 8). **C** Weight of mice during the whole experiment (*n* = 8). **D**–**H** Serum levels of ALT, AST, ALP, TBIL, and γ-GT in mice (*n* = 8). **I** Necrosis index of mice in each group. **J** H&E staining in mice (×100, ×400) (*n* = 3). The red arrow points to the area of hepatic lesion. Data are presented as mean ± SD. *p < 0.05 and **p < 0.01 versus control; #*p* < 0.05 and ##*p* < 0.01, versus APAP

By macroscopic pathological examination, we observed that the APAP mice began to manifest body weight loss compared with the control group after APAP was injected, while the physiological changes were similar in each group in the first 5 days (Fig. 1C). Besides, we also measured the liver index and spleen index of mice, and the results demonstrated that both of them increased in APAP group (*p* < 0.01). By contrast, the HPF and LPF groups could drop them (*p* < 0.01), meaning the swelling of spleen and liver was reduced by PF (Fig. 1A, B).

If hepatocytes are poisoned, infected, necrotized, and inflamed, as a sensitive indicator of liver injury, serum transaminases will be released into the blood, i.e., ALT, AST, and TBIL [27]. We further measured unique indicators of liver functions. Compared with control group, the serum levels of these indices evidently rose in APAP group (*p* < 0.01), noticing that the DILI model had been successfully established. By contrast, PF at high and middle doses significantly diminished the levels of ALT, ALP, γ-GT, TBIL, and AST (*p* < 0.01) compared with the APAP group (Fig. 1D–H). In addition to TBIL (*p* > 0.05), the LPF group (50 mg/kg) was sensitive to the other indicators and effectively reduced their contents (Fig. 1G).

Similar to the biochemical indices, the pathological results showed that hepatic lobules in the APAP group were disorderly arranged, with a large number of necrotic hepatocytes distributed around the portal area and infiltrated with inflammatory factors, besides, focal necrosis were widely observed in the field of view (× 100). Compared with the APAP group, hepatic lobules in the MPF and HPF group were structured and tightly arranged, and the hepatic edema and inflammatory infiltration were improved by PF, especially at the high dose (Fig. 1J). In addition, the necrotic index of hepatic tissues was calculated. The liver injury was relieved after PF treatment (*p* < 0.01) (Fig. 1I). In brief, PF exerted a protective effect on the liver and apparently slowed down the further progress of liver injury induced by APAP.

### PF inhibits ROS to improve the antioxidant power of APAP model mice

**Fig. 2:**
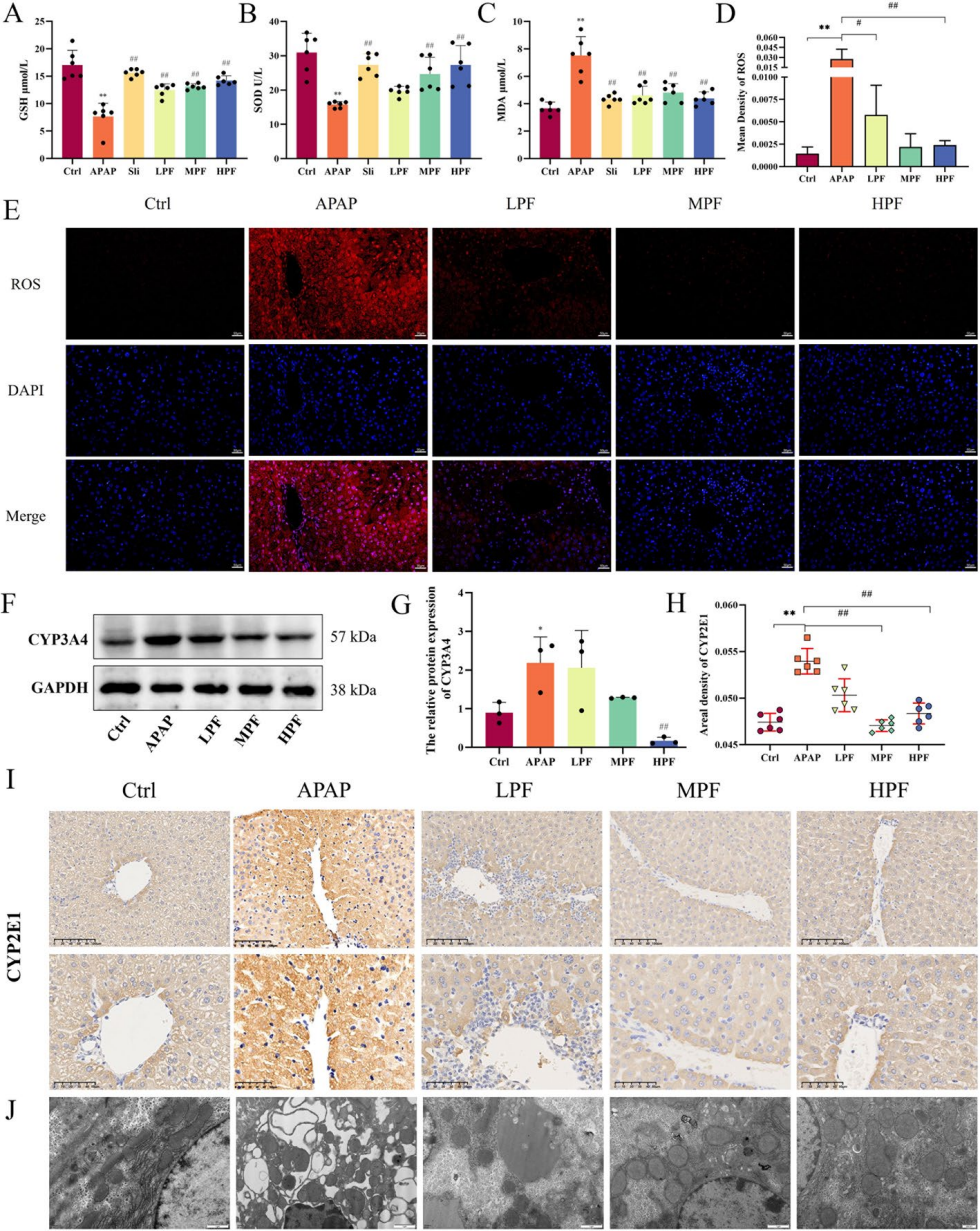
Effects of PF on oxidative stress with APAP stimulation. The levels of GSH (**A**), SOD (**B**), and MDA (**C**) are shown(*n* = 6). **D** Mean density of ROS. **E** Immunofluorescence of ROS (*n* = 3). **F** Western blotting images of CYP3A4 (*n* = 3). **G** Relative protein expression of CYP3A4. **H**, **I** The expression of CYP2E1 by Immunohistochemistry (×200, ×400) (*n* = 3). **J** Transmission electron microscope (*n* = 3). Data are presented as mean ± SD. *p < 0.05 and **p < 0.01, vs. control; #*p* < 0.05 and ##*p* < 0.01, vs. APAP

Some of the crucial signal molecules involved in the oxidative stress response are ROS and antioxidant compounds. In APAP-induced liver injury, the excessive accumulation of ROS disrupts cellular homeostasis and further triggers oxidative stress and mitochondrial dysfunction [28]. To elucidate the capacity of PF treatment on the oxidative status, we evaluated the levels of oxidation-related indicators, such as GSH, SOD, and MDA, at first. Compared with control group, the contents of SOD and GSH were significantly abated, if processed with APAP (*p* < 0.01), while PF restored the antioxidant capacity of the APAP mice at different concentrations, especially at 200 mg/kg (Fig. 2A, B). In contrast to the role of GSH and SOD, changes in the MDA levels occur in response to the oxidative capacity of the body [29]. For MDA, the evident increase occurring in the APAP group and the onset of oxidative effects were mitigated by PF at different doses (*p* < 0.01) (Fig. 2C). These indicators suggested that PF remitted the occurrence of oxidative stress in the APAP model.

Concomitantly, ROS expression was observed and measured by immunofluorescence (Fig. 2E). The IF of ROS showed that the fluorescence expression of ROS in MPF and HPF groups was prominently choked compared to APAP group, suggesting that PF at high and middle doses significantly inhibited ROS expression in hepatocytes (*p* < 0.01) (Fig. 2D). In addition, NAPQI, the metabolite of APAP by the cytochrome P450 enzymes CYP2E1 and CYP3A4, induces major cell death and mitochondrial dysfunction and then leads to damage in the process of accumulation, which means that increased expressions of cytochrome P450 enzymes can be seen in the course of injury by APAP overdoses [30]. Similar results were observed in our experiment. IHC revealed that the positive expression levels of CYP2E1 were higher in the APAP group than in the control and PF groups (Fig. 2I). By contrast, the reactions of CYP2E1 in APAP group predominantly aggravated (*p* < 0.01). However, given PF at doses of 100 and 200 mg/kg overturned overtly the APAP-stimulated oxidative stress compared with the APAP group (*p* < 0.01) (Fig. 2H). Meanwhile, the expression of CYP3A4 was detected by western blotting. The expression of CYP3A4 increased after APAP injection (*p* < 0.05), while this effect was notably reversed by PF at dose of 200 mg/kg (*p* < 0.01) (Fig. 2G). Moreover, the results of TEM exhibited that the mitochondrial structure in APAP group was fuzzy, and the ridge was not clear. The matrix electron density increased and the rough endoplasmic reticulum expanded. After high dose treatment with PF, the mitochondrial structure was complete and the matrix was uniform, which showed a uniform gray structure under the electron microscope. Moreover, the morphological structure of the rough endoplasmic reticulum was restored (Fig. 2J). This evidence explained that PF blocked the generation of ROS, improved the activity of cytochrome P450 enzymes, and enhanced the antioxidant capacity in mice against damage produced by APAP.

### PF refrains the progress of apoptosis on APAP-induced liver injury

**Fig. 3:**
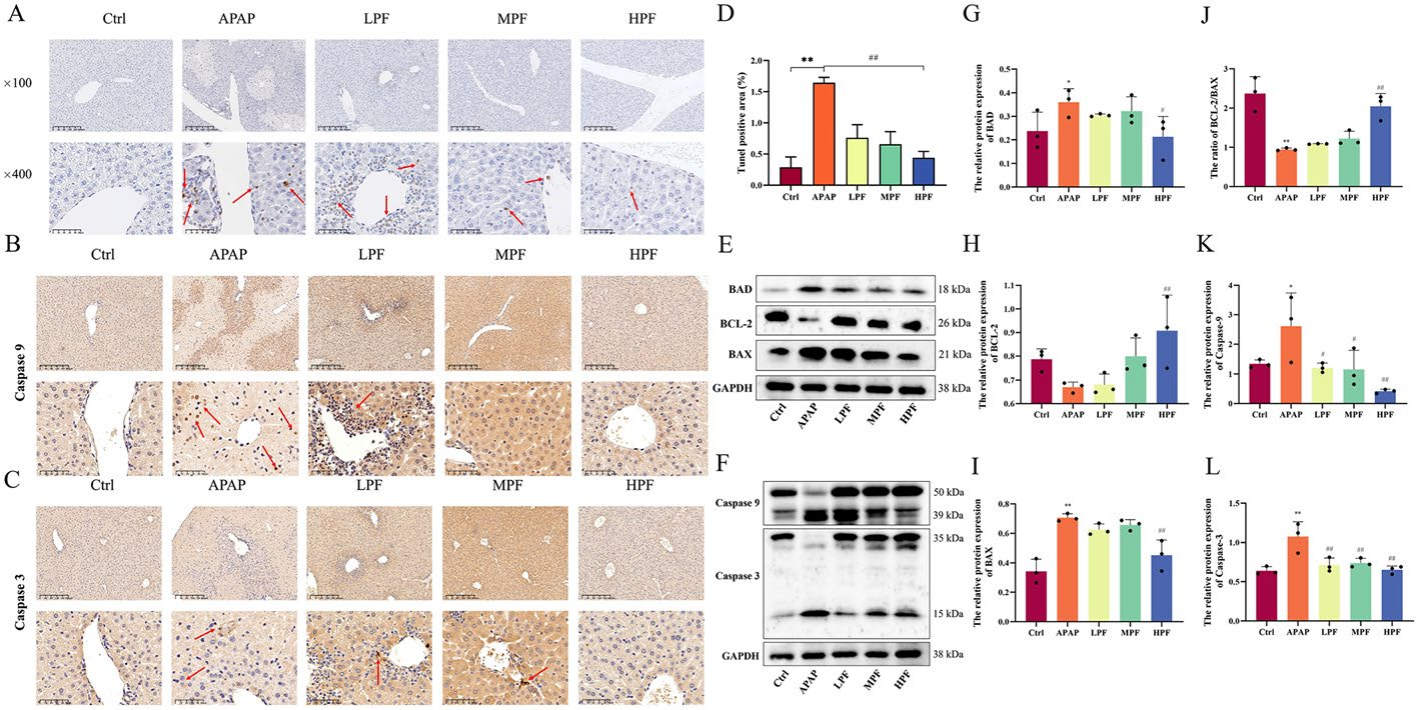
Anti-apoptotic effects of PF in APAP-induced hepatic damage. **A** The apoptotic expression is shown by TUNEL staining (×100, ×400). The expression of caspase 9 (**B**) and caspase 3 (**C**) is shown in the different groups (×100, ×400). **D** The TUNEL positive rate in mice. **E** Western blotting images of BAD, BCL-2, and BAX in mice. **F** Western blotting images of caspase 9 and caspase 3 in mice. The relative protein expression of BAD (**G**), BCL-2 (**H**), BAX (**I**), caspase 9 (**K**), and caspase 3 (**L**) is depicted. **J** Ratio of BCL-2/BAX in mice. The red arrow points to positive expression areas. Data are presented as mean ± SD in each three samples in group. *p < 0.05 and **p < 0.01, versus control; #*p* < 0.05 and ##*p* < 0.01, versus APAP

Along with variation in the mitochondrial outer membrane permeability caused by ROS accumulation, the Bcl-2 protein family releases pro-/anti-apoptotic factors to control the apoptosis process and then activates caspase 9 to further release caspase 3, triggering apoptosis [31]. The TUNEL staining, visually reflected the occurrence of apoptosis, is exhibited in Fig. 3A. Compared with the control group, plenty of positive apoptotic factors gathered in the liver injury area in APAP group. Moreover, compared with APAP group, the positive expression rate of apoptosis in the treatment group was evidently reduced (*p* < 0.01), suggesting that PF improved apoptosis in APAP-induced liver injury (Fig. 3D). To further confirm the role of apoptosis in PF treatment for liver damage, IHC was used to examine the positive expression of apoptotic characteristic factors. The positive expression was concentrated in the hepatic portal area, and the expression of caspase 3 in mice livers stimulated by APAP was more severe, suggesting that the process of liver injury was accompanied by apoptosis. Likewise, the caspase 9 expression displayed low levels in control group and high expression in APAP group. PF pretreatment abated the positive expression of pro-apoptotic proteins (caspase 9 and caspase 3) with an optimal effect appearing at a dose of 200 mg/kg (Fig. 3B, C).

The relative expression of pro-/anti-apoptotic proteins (BAD, BAX, caspase 9, caspase 3, and BCL-2) were assessed by western blotting (Fig. 3E, F). The relative protein expressions of BAD, BAX, and caspase 9 were similar. All of them showed that results were elevated upon the administration of APAP, while the relative expressions of them were distinctly lessoned upon PF exposure (*p* < 0.05 or *p* < 0.01) (Fig. 3G, I, K). Caspase 3 was the most sensitive indicator of apoptosis, and different doses of PF were effective in suppressing the increased expression caused by APAP (*p* < 0.01) (Fig. 3L). Although the result for BCL-2 displayed no difference between the control group and APAP group, an obvious decline in the ratio of BCL-2/BAX was found in APAP group (*p* < 0.01) compared with the control group, whereas an evident rise occurred in the HPF group (*p* < 0.01) (Fig. 3H, J). This result indicates that PF promoted the activity of anti-apoptotic proteins and terminated the expression of pro-apoptotic proteins to exert anti-apoptotic effects.

### Activation of autophagy by PF protects liver from APAP damage

**Fig. 4:**
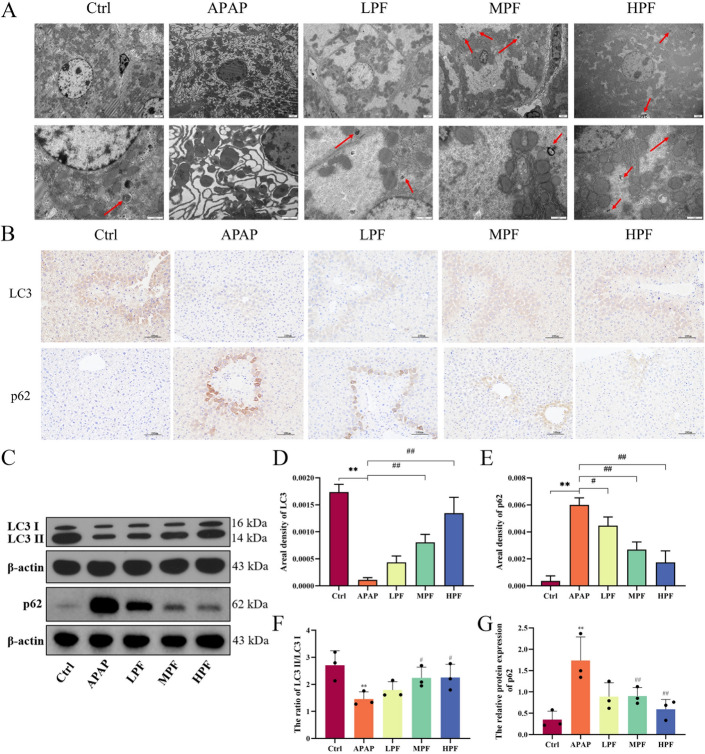
Changes in autophagy representative indices modulated by PF. **A** Transmission electron microscopy of autophagosomes. **B** Immunohistochemistry of LC3 and p62 in different groups (×200). **C** Western blotting images of LC3 and p62. The mean density of LC3 (**D**) and p62 (**E**) in immunohistochemistry. **F** Ratio of LC3II/LC3I in mice. **G** Relative protein expression of p62. The red arrow points to autophagosomes. Data are presented as mean ± SD in each three samples in group. *p < 0.05 and **p < 0.01, versus control; #*p* < 0.05 and ##*p* < 0.01, versus APAP

Relevant studies suggested that autophagy selectively removes impaired mitochondria and NAPQI-protein adducts, thereby blocking the progression of liver damage generated by APAP [32]. TEM was used to observe the number of autophagosomes and the structure of organelles in hepatic tissues (Fig. 4A). Compared with other groups, the rough endoplasmic reticulum in APAP group was extensively swollen and only a few autophagosomes were found. At the same time, apoptosis, nuclear shrinkage, and chromatin concentration were also observed in hepatocytes. Relatively, the number of autophagosomes in PF groups was markedly reinforced, the morphology and structure of hepatocytes were intact, and the chromatin was evenly distributed. LC3, as a downstream manifestation molecule of the autophagy process represents the onset of autophagy by its conversion from type I to type II [33]. Moreover, as the pivot, p62 is closely related to the regulation of autophagy and mitochondrial autophagy [34]. Thus, IHC and WB were used to visualize changes in proteins (LC3 and p62) associated with autophagy regulation (Fig. 4B, C). The results of IHC displayed that PF increased the positive expression of LC3 (*p* < 0.01), exerting the promoting effect for autophagy (Fig. 4D). On the other hand, the expression of p62 was remarkably boosted in APAP group, while this was alleviated by PF treatment (*p* < 0.01) (Fig. 4E). Similar to IHC, the results of WB showed an evidently inhibition of p62 expression in the MPF and HPF groups (*p* < 0.01). For LC3, the ratio of type II to type I was obviously reduced by APAP (*p* < 0.01), and PF effectively mitigated this process (*p* < 0.05) (Fig. 4F, G).

**Fig. 5:**
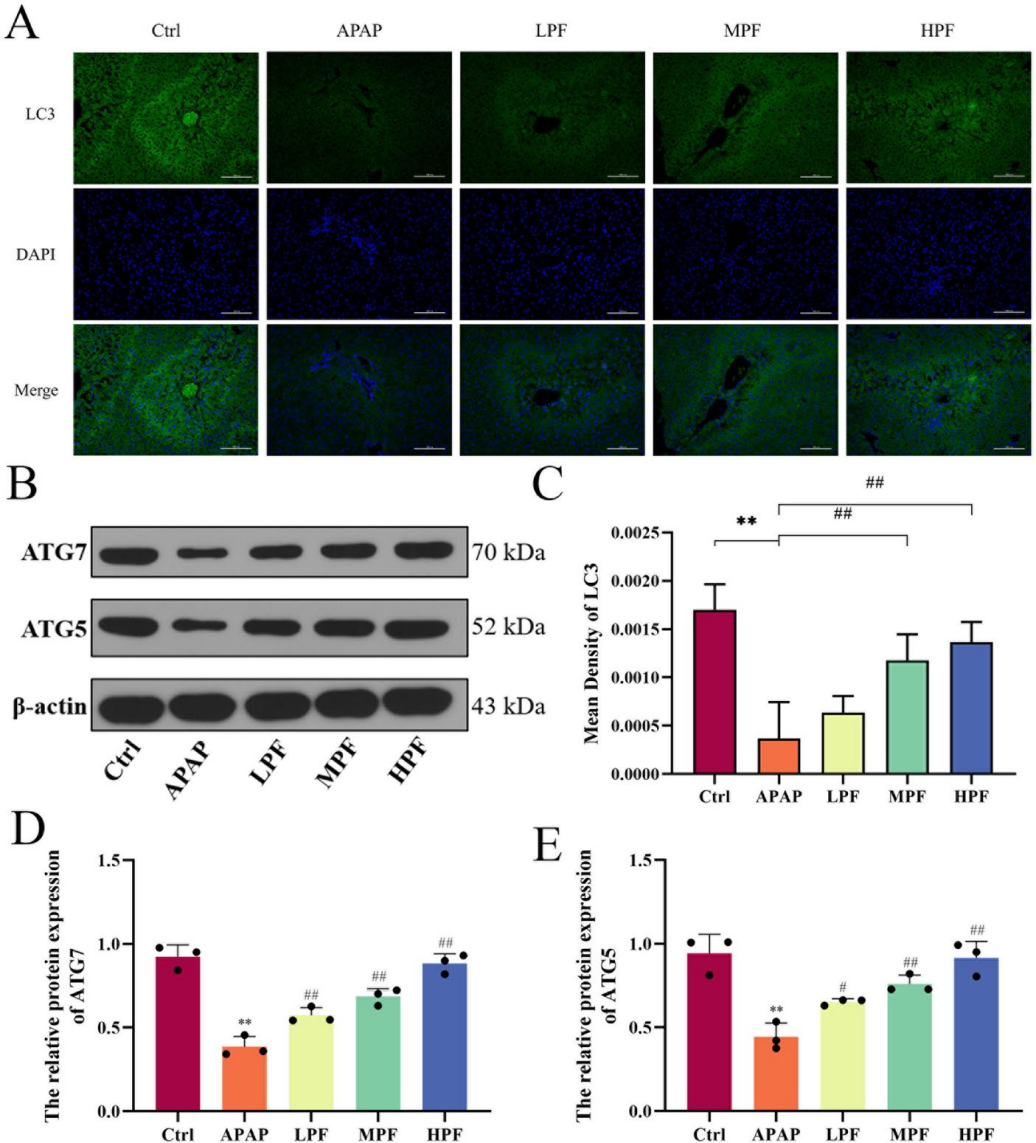
Activation of autophagy by PF on APAP-induced mice. **A** Immunofluorescence of LC3. **B** Western blotting images of ATG7 and ATG5. **C** Mean density of LC3 in immunofluorescence. Relative protein expression of ATG7 (**D**) and ATG5 (**E**). Data are presented as mean ± SD in each three samples in group. *p < 0.05 and **p < 0.01, versus control; #*p* < 0.05 and ##*p* < 0.01, versus APAP

Furthermore, the immunofluorescence results showed a weak fluorescence in the APAP group and a strong fluorescence in the MPF and HPF groups (*p* < 0.01) (Fig. 5A, C). The results of WB suggested that both ATG5 and ATG7 were depleted in APAP group, compared with control group (*p* < 0.01). Instead, PF reversed the effect of APAP and promoted the expression of them (*p* < 0.05 or *p* < 0.01) (Fig. 5D, E). We also measured mRNA expression levels to determine autophagic activity changes in APAP-induced mice. The levels of *ATG5*, *ATG7*, and *BECN1* in the APAP group decreased (*p* < 0.05 or *p* < 0.01) compared with the control group. More importantly, HPF improved the mRNA expression pattern in the APAP model (*p* < 0.05), despite the fact that the effect was hidden in *ATG7*, and only the LPF group showed an alleviation (Figure S3). These results illustrated that PF effectively remitted the inhibition of autophagy generated by APAP.

### Transcriptomics reveals in-depth mechanisms of PF on APAP-induced hepatic injury

The current findings indicated that the amelioration of APAP-induced DILI by PF was closely related to the activation of autophagy, inhibition of oxidative stress, and apoptosis. Yet, the mechanisms by which PF regulated the role of these phenotypes were not clear. Therefore, we sequenced hepatic samples of mice to further obtain the potential mechanism of PF against liver injury caused by APAP. Data preprocessing suggested homogeneous gene expression in the samples and correlation analysis informed good correlation of samples (*R*^2^ = 0.858–0.994) (Figure S4A, B). Moreover, the results of the principal component analysis (PCA) showed that the samples within the group were consistent and the classification between the groups was obvious, which manifested the prominent discrepancy among these groups and the subsequent differential gene analysis results were feasible (Figure S4C). In summary, the above results indicated that the sequencing data were reliable.

**Fig. 6:**
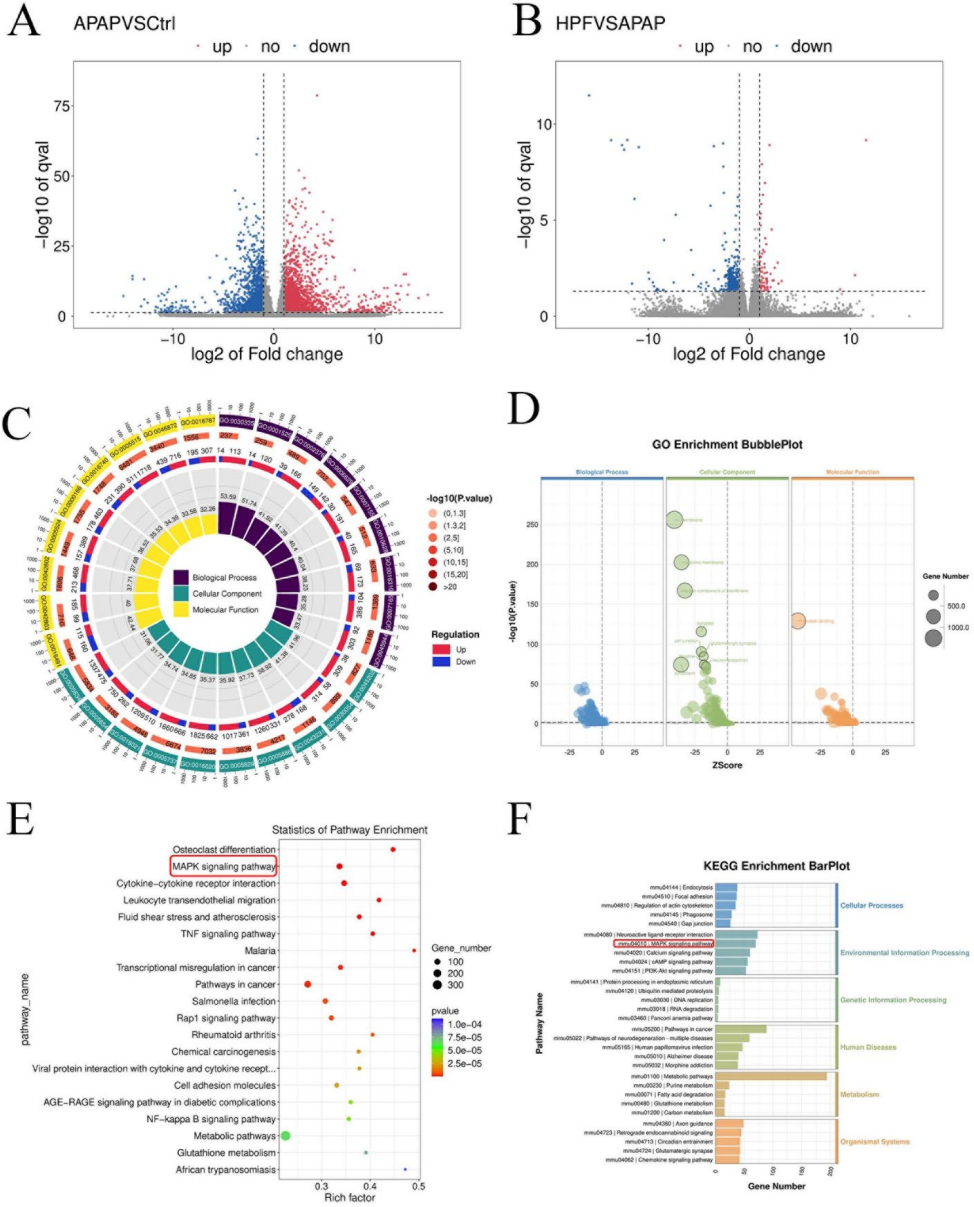
Transcriptomic analysis of PF in improving APAP-induced liver injury. Volcano map of the control versus APAP groups (**A**) and HPF versus APAP groups (**B**). GO analysis of the control versus APAP groups (**C**) and HPF versus APAP groups (**D**). KEGG analysis of control verus APAP groups (**E**) and HPF versus APAP groups (**F**)

Quantitative analysis of gene expression for each group was compared and presented in Figure S4D. Different from the control group, 2444 genes were upregulated and 1552 genes were downregulated in the APAP group. A total of 508 different genes with 443 downregulated and 65 upregulated were presented between the HPF group and the APAP group (Fig. 6A, B). The APAP group clustered in oxidative stress and cell death, whereas the clustered genes of HPF group were less in these area under the cluster analysis (Figure S5). For the gene set enrichment analysis (GSEA), PF exerted regulatory effects on several signals and physiological processes, including MAPK signaling pathway, mTOR signaling pathway, apoptosis, and glutathione metabolism (Figure S6). In addition, combining the results of GO analysis and KEGG enrichment, the MAPK pathway showed the higher score and performed simultaneously in all three sets of differential gene analysis (Fig. 6C–F). All comparisons between the HPF group and the control group appeared in Figure S7. Therefore, the results of the transcriptomics analysis suggested that the MAPK signaling pathway played a key role for PF’s activity on drug-induced liver injury by APAP, which further regulated mTOR signal, then activated autophagy, and inhibited oxidative stress and apoptosis.

### PF blocks MAPK/mTOR signaling pathway by directly targeted p38

**Fig. 7:**
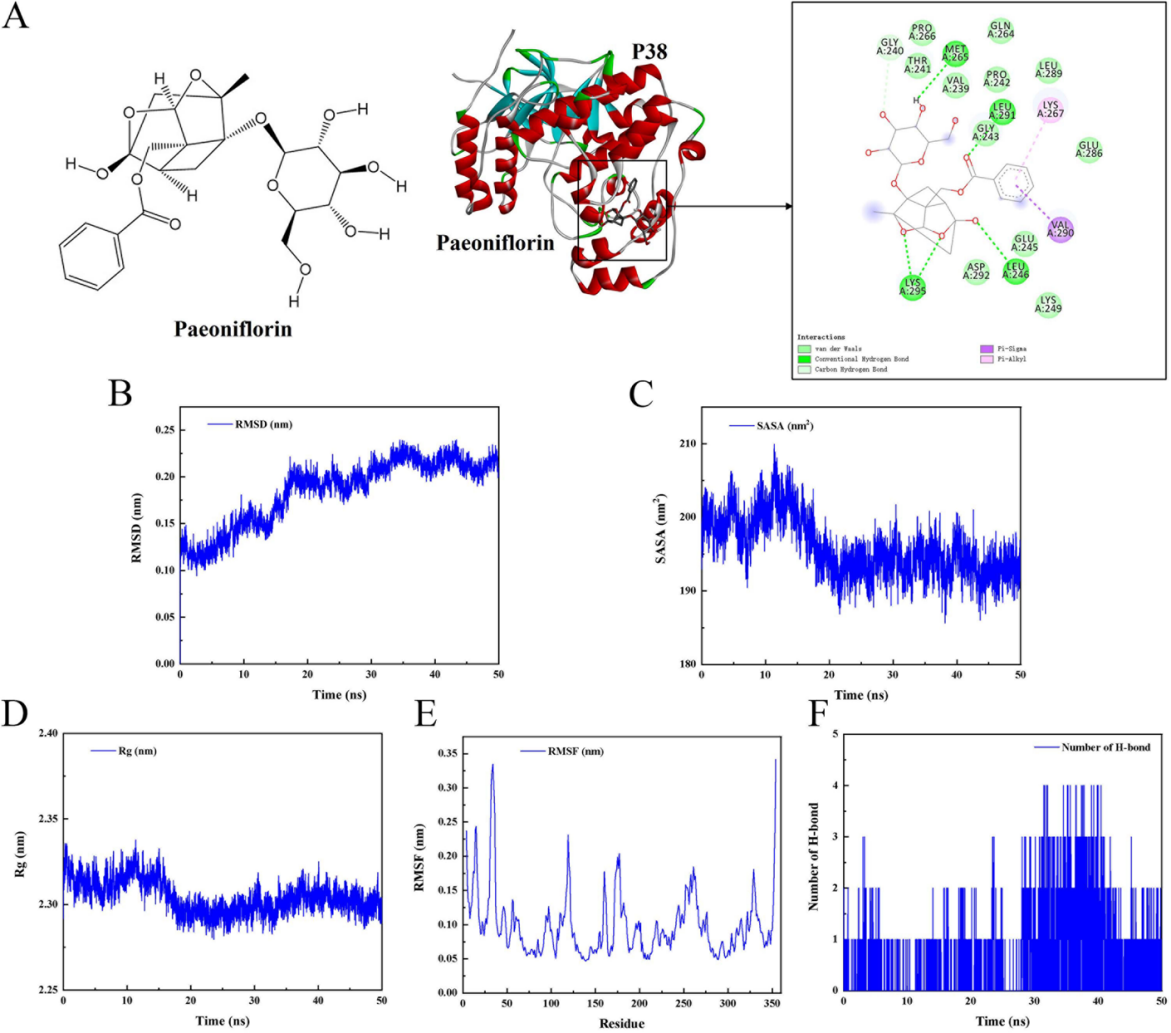
Targeting of p38 by PF. **A** Molecular docking analysis of PF to p38. **B** RMSD of the p38–PF complex. **C** SASA of the p38–PF complex. **D** Rg of the p38–PF complex. **E** RMSF of the p38–PF complex. **F** Hydrogen bonding analysis of the p38-PF complex

Based on the transcriptomic results, we were intrigued about the importance of MAPK signal in DILI development and PF therapy. To further examine the role of PF on MAPK signaling, molecular docking analyses were performed. The p38-PF complex displayed a great binding capacity with a binding energy of − 32.50 ± 0.71 kJ/mol in contrast to the ERK-PF complex with a binding energy of − 21.65 ± 0.98 kJ/mol (Table S5), which suggested that p38 might be a potential target for PF. PF was bound to the MET265, LEU291, and LEU246 residues via one hydrogen bond, respectively, while LYS295 was connected with two hydrogen bonds to PF. In addition, GLY240 was bound by one carbon hydrogen bond to PF (Fig. 7A). Subsequently, the binding ability of PF to p38 was explored by molecular dynamics simulations. The root mean square deviation (RMSD) results showed that the protein–ligand complex reached the equilibrium after 20 ns, indicating that the entire simulation was stable and reliable (Fig. 7B). Both solvent-accessible surface area (SASA) and radius of gyration (Rg) were reflected the tightness of the complex protein structure during simulation, and our results indicated a decreasing trend in both variables, suggesting an increase in protein tightness structure and a good combination of p38-PF complex (Fig. 7C, D). The protein root mean square fluctuation (RMSF) of the p38-PF complex were less than 0.35 nm, showing a stable protein–ligand binding (Fig. 7E). Besides, the hydrogen bonding analysis suggested that the average hydrogen bond number distribution of p38 to PF was 0.51 (Fig. 7F). Meanwhile, the results of the binding energies of p38 to PF listed in Table 1. The total binding free energy was −79.263 kJ/mol, which indicated a remarkable stability in the p38-PF complex, and the main interactions were given by van der Waals forces and electrostatic energy. These results suggested that PF may target p38 rather than ERK in the MAPK signal cascade and that the binding between PF and p38 was stable.

**Table 1:** Molecular Mechanics Poisson-Boltzmann Surface Area (MMPBSA) analysis of the p38-PF complex

Energy	Protein–ligand (kJ/mol)
Van der Waals energy	−147.133
Electrostatic energy	−72.562
Polar solvation energy	153.000
Nonpolar solvation energy	−18.268
Total binding energy	−152.475
T∆S	−84.963
Total binding free energy	−79.263

**Fig. 8:**
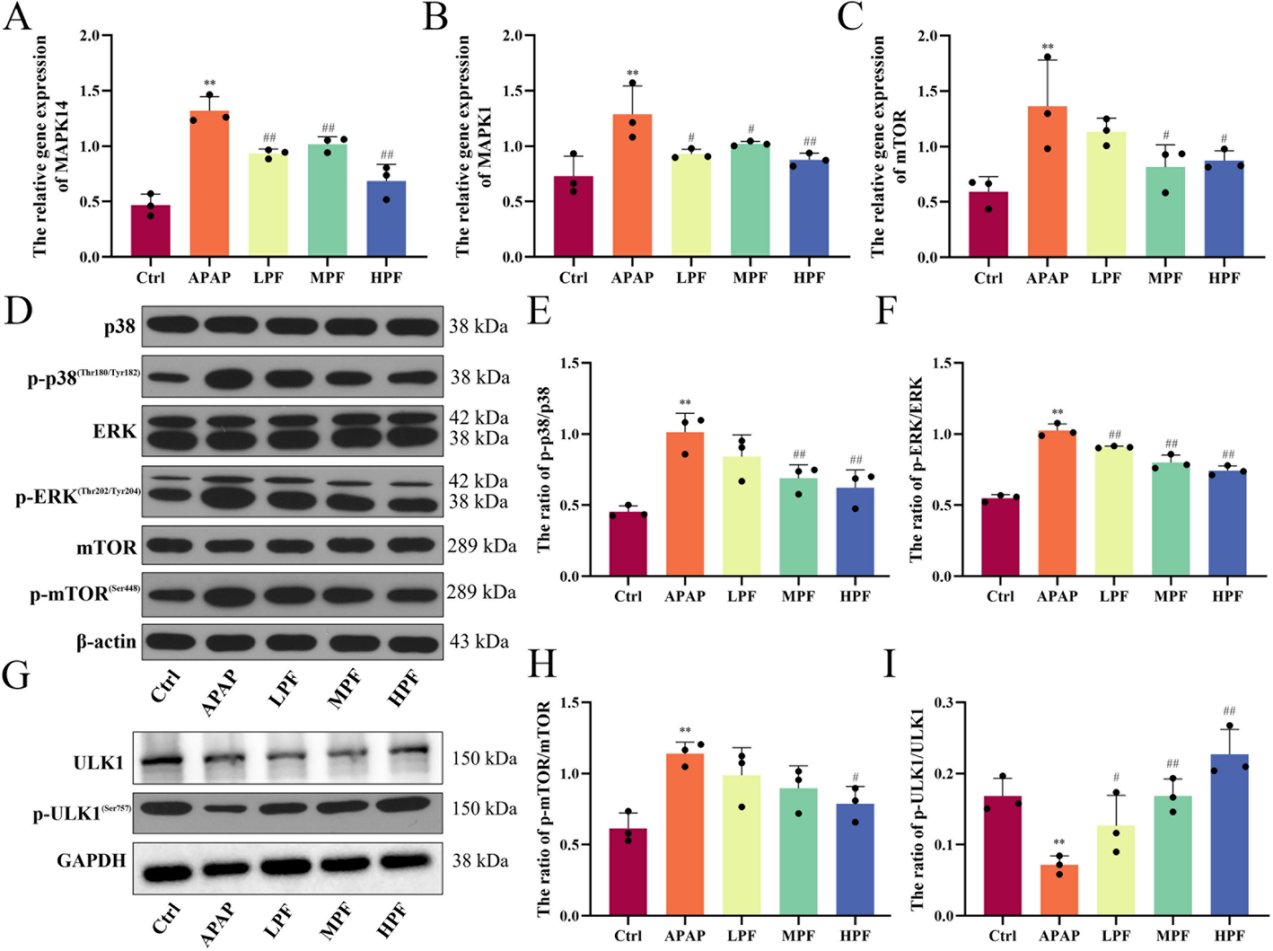
Regulation of PF on MAPK/mTOR signaling. **A**–**C** Relative gene expression of *MAPK14*, *MAPK1* and *MTOR*. **D** Western blotting images of p38, p-p38, ERK, p-ERK, mTOR, and p-mTOR. **E**, **F** Ratio of p-p38/p38 and p-ERK/ERKin mice. **G** Western blotting images of ULK1 and p-ULK1. **H**, **I** Ratio of p-mTOR/mTOR and p-ULK1/ULK1 in mice. Data are presented as mean ± SD in each three samples in group. *p < 0.05 and **p < 0.01, versus control; #*p* < 0.05 and ##*p* < 0.01, versus APAP

Furthermore, the results of mRNA expression were consistent with the transcriptome analysis. PF (200 mg/kg) dramatically downregulated the relative expression of *MAPK1*, *MAPK14*, and *mTOR* (*p* < 0.05 or *p* < 0.01) (Fig. 8A–C). The western blotting results manifested that phosphorylation of p38 and ERK proteins were significantly activated after APAP overdose injection, whereas MPF and HPF effectively inhibited this change (*p* < 0.01) (Fig. 8E, F). However, for phosphorylation of mTOR, the inhibition was only shown at a concentration of 200 mg/kg PF (*p* < 0.05) (Fig. 8H). This suggested that PF acted on MAPK signaling which in turn regulated the phosphorylation of mTOR. Besides, the phosphorylation of ULK1 was inhibited by APAP stimulation, whereas PF memorably reversed this effect and restored the phosphorylation of ULK1, which further activated the downstream autophagy proteins (*p* < 0.05 or *p* < 0.01) (Fig. 8G, I). In brief, the results revealed that PF initiated autophagy through blocking MAPK/mTOR signaling to protect hepatocytes.

## Discussion

As the number of patients with liver diseases is tremendously growing, abundant reports have focused on the role of natural products in liver diseases, especially in drug-induced liver injury [17, 35]. However, due to the extended incubation period, swift onset, and absence of established clinical medications, there is a need for comprehensive investigations into the mechanisms of potential agents in the treatment of DILI. Paeoniflorin has gained widespread attention since its first extraction, and a large number of researches have reported its effects in the digestive, nervous, and cardiovascular systems [36, 37]. Previous studies have focused on the relationship between PF and inflammation and found that PF modulated the Th1/Th2 cytokine balance and promoted the restoration of the inflammatory microenvironment in lichen planus [38]. Based on chemical biology experimental tools, relevant studies have confirmed that PF targeted C1qa to promote colonic inflammation and bacterial clearance [39]. The present study, for the first time, focused on the regulatory role of PF in cell death and delved into the specific mechanism of PF to improve APAP-induced liver injury, confirming that PF exerted protective effects in hepatocytes by downregulating p38 to silence MAPK/mTOR signal, which in turn activated autophagy and inhibited oxidative stress and apoptosis.

According to our study, the loss of weight in APAP mice was recovered by PF, as well as the edema of liver and spleen, which provided evidence for PF treating DILI. Herein, the pharmacodynamics evaluation was consistent with the previous study that PF significantly ameliorated the liver function indices and alleviated the pathological lesions such as inflammatory infiltration in APAP mice. Therefore, we further explored the protective mechanisms of PF on APAP-induced liver injury. Cell survival and death are widely involved in the process of APAP-induced liver injury [40, 41]. Currently, related modes of programed cell death were reported in DILI, including apoptosis, autophagy, necrosis, ferroptosis, and pyroptosis [42, 43]. Among them, autophagy as a high conserved cellular process is thought to be critical in affecting cell survival. Hepatocytes are dependent on basal autophagy to supple energy because of the high biosynthetic activity and carbohydrate storage. Furthermore, the activation of autophagy happened in liver with protein adducts aggregation, oxidative stress, inflammation, and lipid accumulation [29, 44]. In APAP-induced liver injury, autophagy improves liver injury by the selective removal of APAP-protein adducts and damaged mitochondria. It is noteworthy that microstructural experiments found that autophagosomes formed a unique band pattern around the necrotic region after APAP injury to restrict the progression of necrosis. Another study suggested that the inhibition of autophagy in the early stages of APAP injury caused the production of highly reactive APAP adducts [10, 45, 46]. Our findings provide evidence that PF treatment enhanced the expression of ATG5 and ATG7 in APAP mice and promoted the conversion of LC3 type I to type II, supporting the view that PF enhance autophagy to protect hepatocytes during APAP injury.

On the other hand, the occurrence of apoptosis in APAP-induced liver injury has been controversial. In fact, APAP-intoxicated hepatocytes do not show the typical apoptotic cell characteristics and remind more of necrotic and lysogenic death [47]. However, it is undeniable that apoptosis occurs at least in the initial stages of APAP injury and transitions to secondary necrosis thereafter [48]. The production of APAP adducts leads to the over-activation of JNK, which in turn triggers mitochondrial dysfunction, promotes the release of pro-apoptotic factors such as Bcl-2 family members, and then activates the downstream caspase cascade causing apoptosis [49]. Thus, we observed apoptotic factors in the necrotic region of hepatocytes by TUNEL staining. The expressions of pro-apoptotic proteins in APAP mice were evidently upregulated as detected by IHC and WB. In comparison, PF was shown the significant anti-apoptotic effects by regulating the balance of pro-apoptotic and anti-apoptotic proteins. In addition, we found that APAP injury was accompanied by the accumulation of ROS, which indicating the occurrence of oxidative stress. Whereas the regulation of ROS by PF was bidirectional, on the one hand, in cancer cells, it ameliorates gastric cancer by boosted downstream apoptosis through mediated Nox4-regulated ferroptosis [50]. By contrast, alleviating oxidative stress in hepatocytes by attenuating ROS release helps protect hepatocytes from apoptotic damage. Moreover, we further confirmed the occurrence of oxidative stress in APAP mice by measuring GSH, SOD, and MDA. Besides, we examined the activity of the cytochrome P450 enzymes and showed that PF reduced the expression of CYP2E1 and CYP3A4. Cytochrome P450 enzymes are participated in the metabolism of drugs as a major enzyme in the second phase of hepatic metabolic reactions. Among them, several studies have demonstrated the importance of CYP2E1 in drug-induced hepatotoxicity [51–53]. It promotes the accumulation of NAPQI in the liver, exacerbates the mitochondrial burden to release excess ROS, ultimately causing oxidative stress [54]. It is reported that blocking autophagy could trigger the generation of ROS to launch apoptosis, which in turn leads to cell death [55]. Our findings also indicate that the activation of oxidative stress further regulates apoptosis to damage hepatocytes, and the initiation of autophagy launched by PF treatment is effective in mitigating ROS generation and inhibiting oxidative and subsequent apoptosis.

**Fig. 9:**
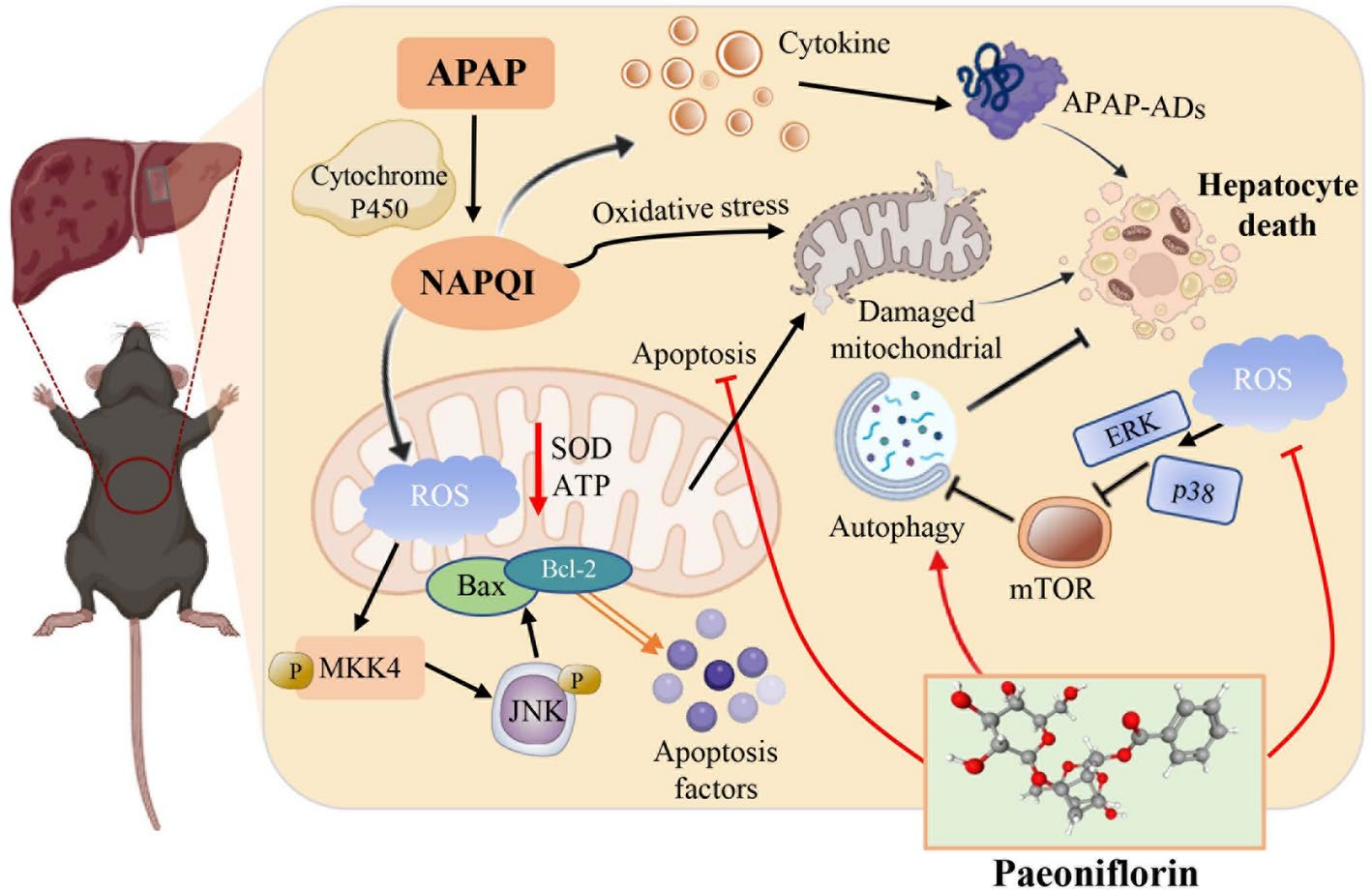
Schematic diagram of PF treatment on hepatic injury with APAP stimulation. Black arrows represent the development of hepatic injury generated by APAP, while red arrows suggest the mechanism of action of paeoniflorin in ameliorating APAP-induced liver injury

The MAPK pathway always played a vital role in controlling cell growth, differentiation, apoptosis, and response to stress and immune [56]. And, mTOR is a key target for regulating ULK1 kinase activity and exerts negative autophagy regulation by directly inhibited ULK1 phosphorylation [33, 57]. Signal crosstalk between the MAPK cascade and mTOR is involved in regulating the initiation and closure of autophagy, modulating the balance between cell proliferation and differentiation as well as occurrence of inflammation and apoptosis [58–60]. These facts are consistent with our transcriptomics results, which revealed that MAPK signaling was upregulated in the APAP gene sequence, while it was suppressed by PF. The combination of predictive tools such as machine learning and molecular experimental validation helps to obtain key targets of PF regulation [61]. And the results of WB and PCR showed that PF distinctly inhibited the phosphorylation of ERK and p38, whereas molecular docking suggested a lower binding energy of PF to p38 compared with PF to ERK. The dynamic interaction of PF to p38 was further studied by molecular dynamics simulations, which displayed a stable binding. It is hypothesized that p38 is a core target for the action of PF. In short, the mechanistic studies have demonstrated that PF counteracted APAP-induced liver injury by activating autophagy, restraining apoptosis and oxidative stress through downregulation of the MAPK/mTOR pathway (Fig. 9). Although this study has made important discoveries and breakthroughs, some shortcomings and limitations are still remained. One point worthy to discuss is that the current precise crosstalk between MAPK regulation and autophagy is still unclear, and the balanced relationship between autophagy and apoptosis remains largely controversial. Besides, experiments with small samples increase the bias and inconsistency of results. Hence, in future research, subsequent large-scale, multicenter studies should be conducted to further validate and expand the findings of this study.

## Conclusions

To sum up, our work suggested that paeoniflorin inhibited the key process of APAP-induced liver injury and thus exerted a restorative effect. We also noticed that the protective effects of paeoniflorin were achieved by the induction of autophagy and the containment of apoptosis and oxidative stress through targeting p38 to downregulate MAPK/mTOR signaling. Despite the existence of some limitations, the results of this work further fill the gap of knowledge for paeoniflorin in the prevention and treatment of DILI, which may help to advance the development of clinical applications of paeoniflorin.

## Supplementary Information

Supplementary Material 1.

## Data Availability

The core data for this work has been supplied in the manuscript. Further data will be made available upon reasonable request.

## Abbreviations

BECN1: Beclin 1
ATG5: Autophagy-related 5
ATG7: Autophagy-related 7
ALP: Alkaline phosphatase
ALT: Alanine transaminase
APAP: Acetaminophen
AST: Aspartate transaminase
BSA: Bovine serum albumin
CYP3A4: Cytochrome P450 family 3 subfamily A member 4
CYP2E1: Cytochrome P450 family 2 subfamily E member 1
DAB: 3,3′-Diaminobenzidine
DILI: Drug-induced liver injury
GST: Glutathione
H_2_O_2_: Hydrogen peroxide
IF: Immunofluorescence
IHC: Immunohistochemistry
mTOR: Mammalian target of rapamycin
MAPK: Mitogen-activated protein kinase
MDA: Malondialdehyde
NAC: *N*-Acetylcysteine
NAPQI: *N*-Acetyl-*p*-benzoquinone imine
NSAID: Nonsteroidal anti-inflammatory drugs
PBS: Phosphate-buffered saline
PF: Paeoniflorin
PMSF: Phenylmethylsulfonyl fluoride
Rg: Radius of gyration
RIPA: Radioimmunoprecipitation assay
RMSD: Root mean square deviation
RMSF: Root mean square fluctuation
ROS: Reactive oxygen
SASA: The solvent-accessible surface areas
SD: Standard deviation
SOD: Superoxide dismutase
TBIL: Total bilirubin
TEM: Transmission electron microscopy
TUNEL: Terminal deoxinucleotidyl transferase (TdT)-mediated dUTP-fluorescein nick and labeling
WB: Western blot
γ-GT: γ-Glutamyl transpeptidase
