# Supplementary material for: Paeoniflorin protects hepatocytes from APAP-induced damage through launching autophagy via the MAPK/mTOR signaling pathway
Source: Cell Mol Biol Lett. 2024 Sep 7;29:119. doi: 10.1186/s11658-024-00631-4 (PMC11380789; doi:10.1186/s11658-024-00631-4)
Supplement: Supplementary file 1 — Supplementary Material 1. [file 11658_2024_631_MOESM1_ESM.doc]

**Paeoniflorin guards hepatocyte from APAP-induced damage through launching autophagy via MAPK/mTOR signaling pathway**

*Xinyu Deng^a^, Yubing Li^a^, Yuan Chen^a^, Qichao Hu^a^, Wenwen Zhang^a^, Lisheng Chen^a^, Xiaohua Lu^c*^, Jinhao Zeng^b*^, Xiao Ma^a*^, Thomas Efferth^c*^*

*^a^State Key Laboratory of Southwestern Chinese Medicine Resources, School of Pharmacy, Chengdu University of Traditional Chinese Medicine, Chengdu, 611137, China*

*^b^TCM Regulating Metabolic Diseases Key Laboratory of Sichuan Province, Hospital of Chengdu University of Traditional Chinese Medicine, Chengdu, China*

*^c^Department of Pharmaceutical Biology, Institute of Pharmaceutical and Biomedical Sciences, Johannes Gutenberg University, Mainz, Germany*

^*^ Corresponding authors: Xiaohua Lu: xiaohulu@uni-mainz.de; Jinhao Zeng, Email address: zengjinhao@cdutcm.edu.cn; Xiao Ma, Email address: tobymaxiao@cdutcm.edu.cn; Thomas Efferth, E-mail address: efferth@uni-mainz.de

**Supplementary File**


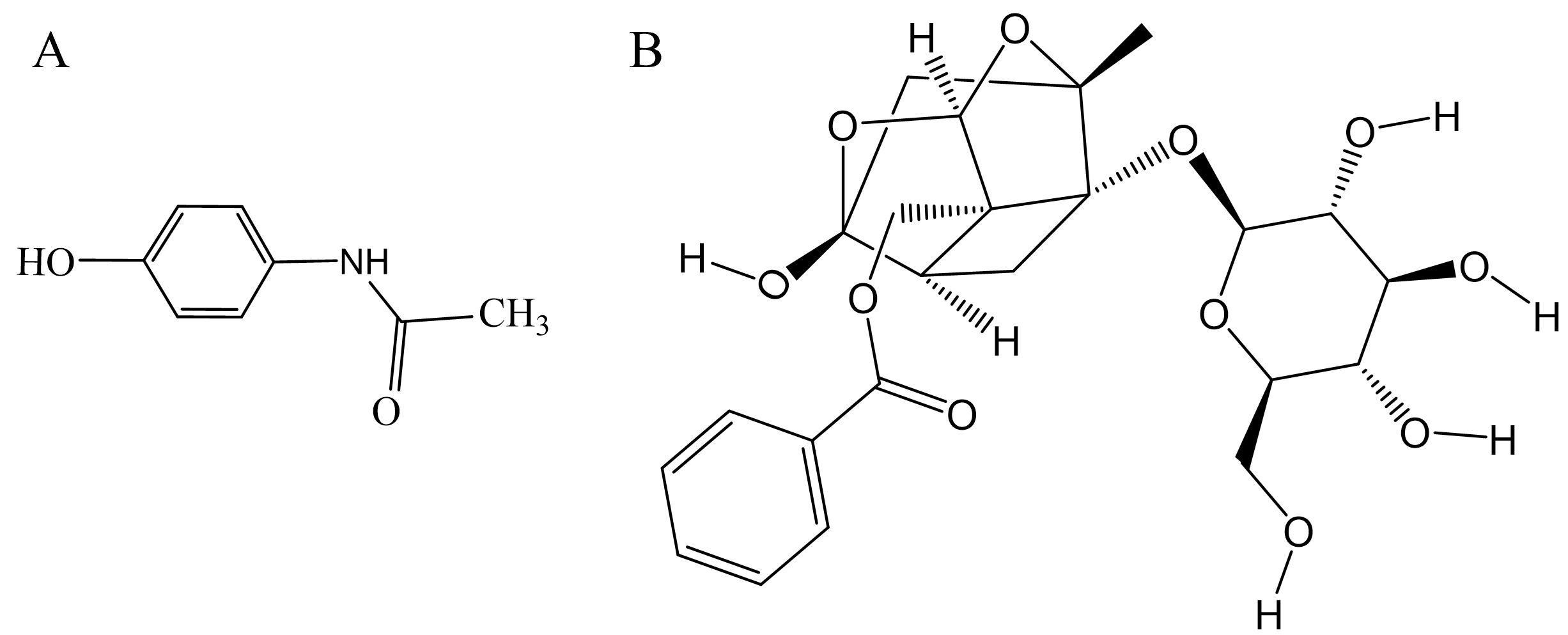


**Figure S1 The chemical structures.**

**(A) the structure of acetaminophen (B) the structure of paeoniflorin**





**Figure S2 Experimental procedure of PF against APAP-induced liver injury**

1. **Experimental procedure of control group (B) Experimental procedure of model group (C) Experimental procedure of positive group (D) Experimental procedure of treatment group**


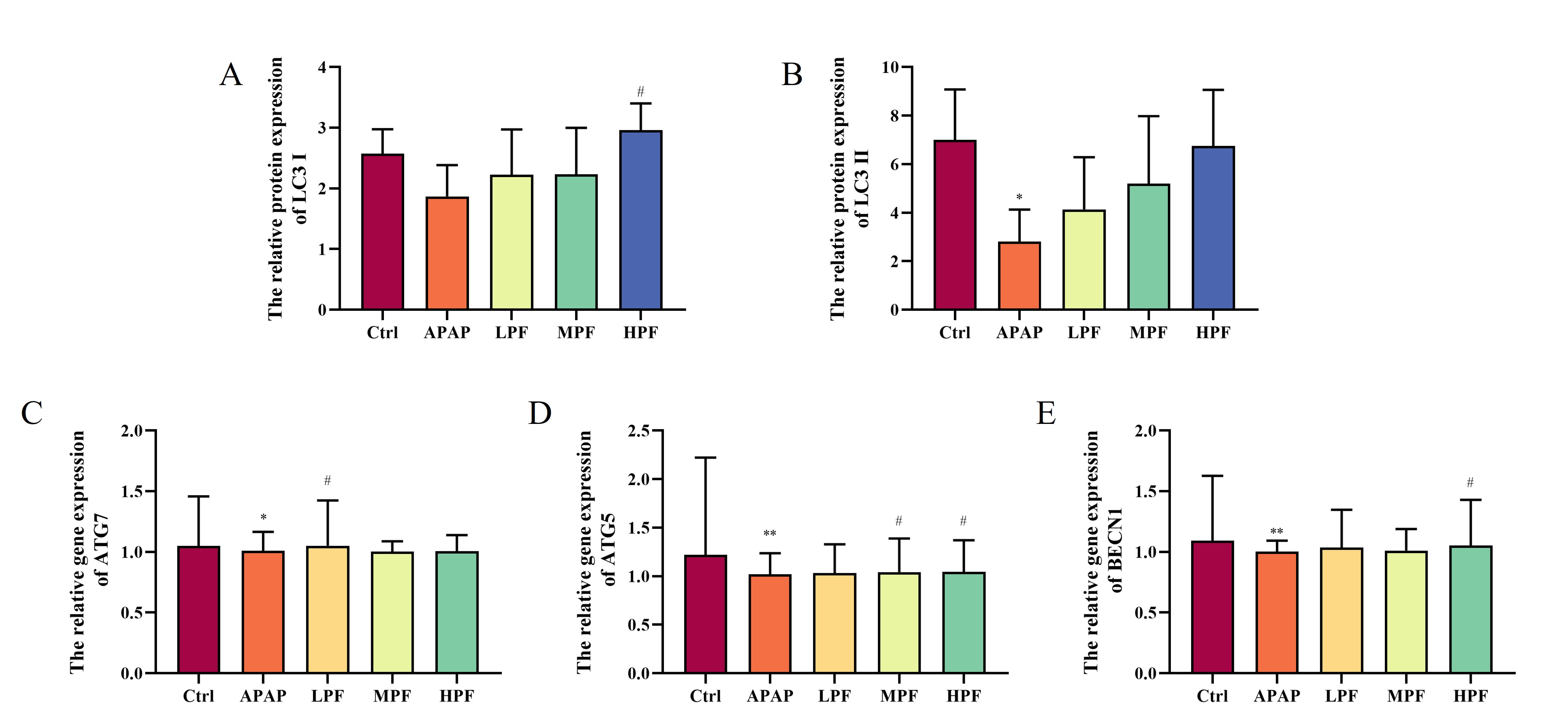


**Figure S3 Effects of PF on autophagy proteins and genes**

**(A-B) the relative protein expressions of LC3 I and LC3 II (C-E) the relative gene expression of ATG7, ATG5 and BECN1. Data are presented as mean ± SD in each three samples in group. ^*^*p* < 0.05 and ^**^*p* < 0.01, vs. control; ^#^*p* < 0.05 and ^##^*p* < 0.01, vs. APAP**


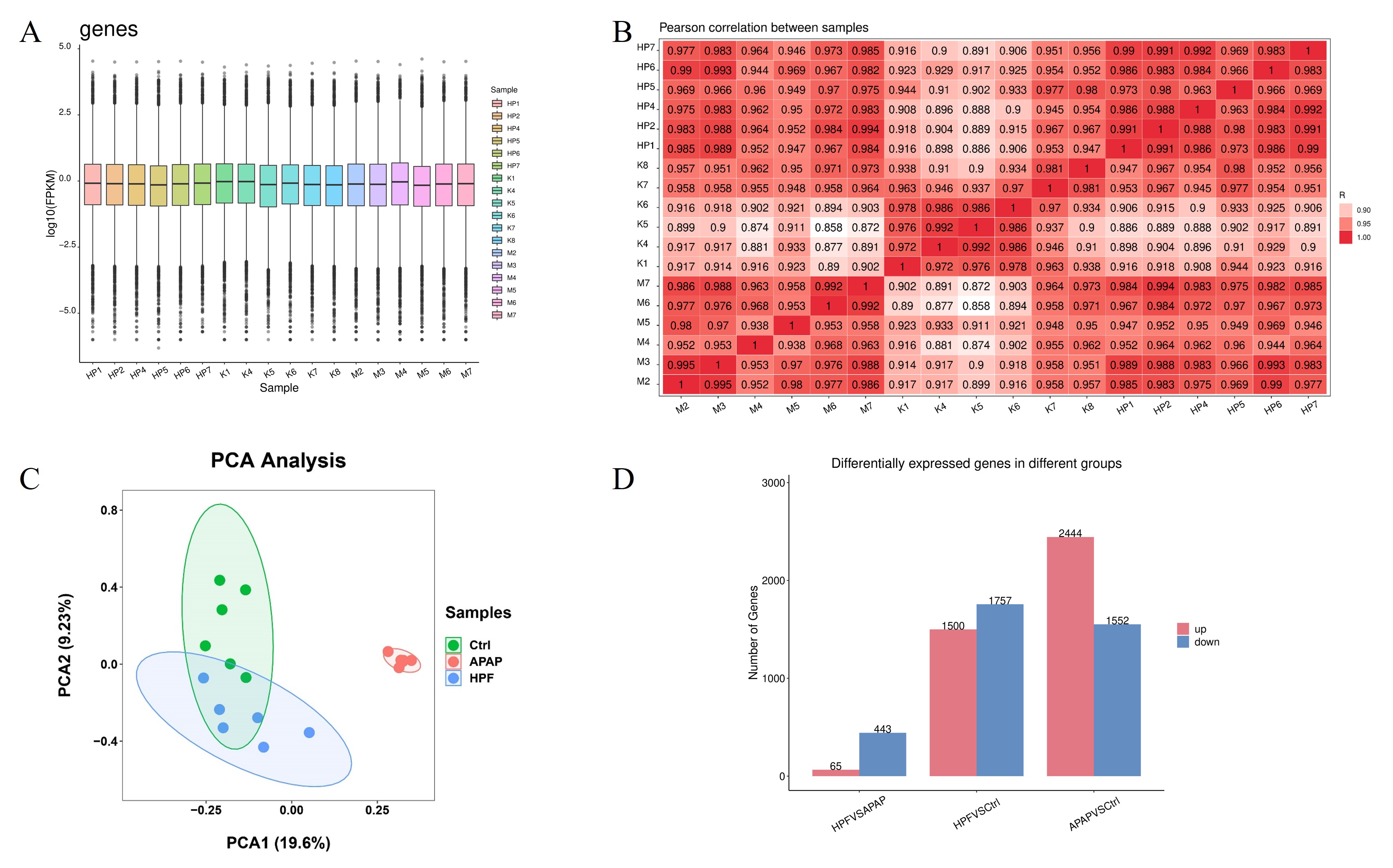


**Figure S4 Data preprocessing of transcriptome data analysis**

**(A) the gene expression of samples (B) Pearson correlation coefficient analysis (C) The PCA analysis of samples (D) Quantitative analysis of gene expression**

**

**

**Figure S5 The cluster analysis of samples**

**(A) the cluster analysis of Ctrl group to APAP group (B) the cluster analysis of HPF group to APAP group**

**
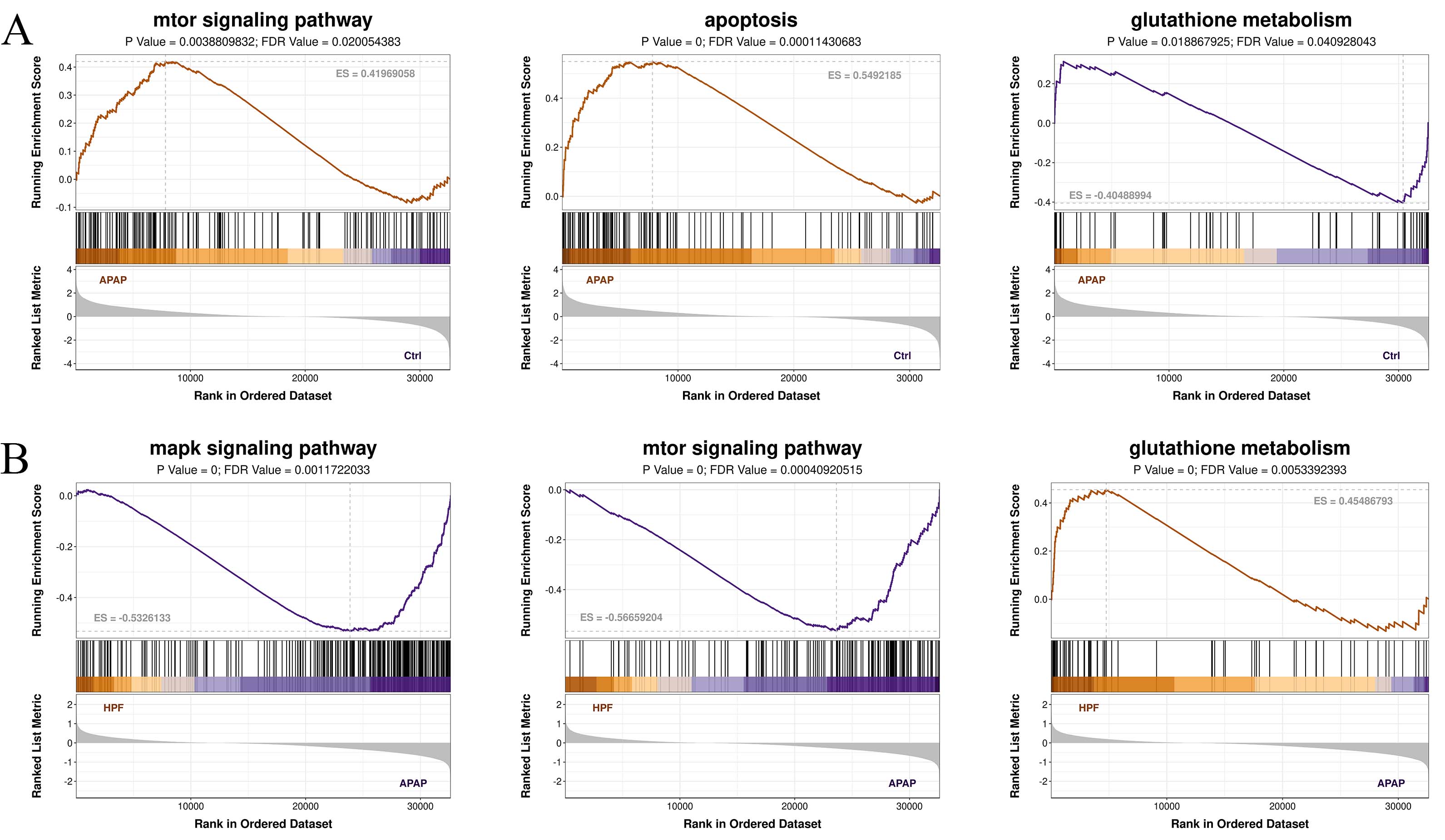
**

**Figure S6 The gene set enrichment analysis**

**(A) the gene set enrichment analysis of Ctrl group and APAP group (B) the gene set enrichment analysis of HPF group and APAP group**

**

**

**Figure S7 The details of compare in HPF group and Ctrl group**

1. **the cluster analysis of Ctrl group to HPF group (B) The volcano map of Ctrl VS HPF (C) The GO analysis of Ctrl VS HPF (D) The KEGG analysis of Ctrl VS HPF**

**
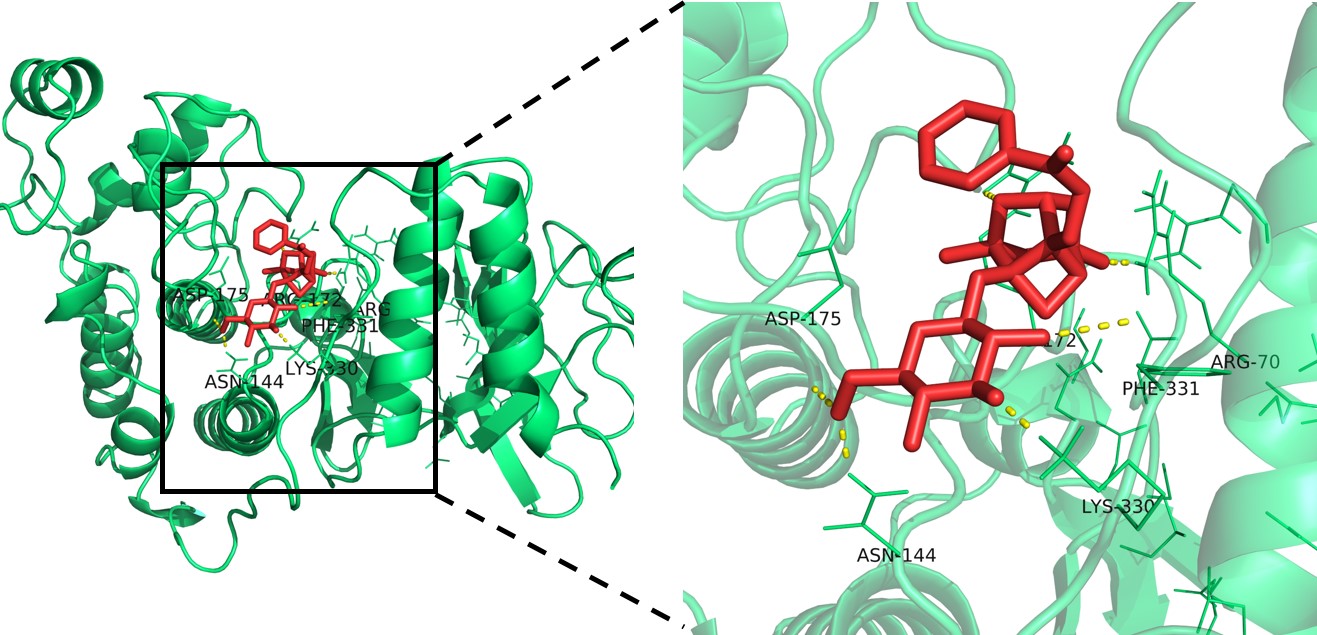
**

**Figure S8 The molecular docking analysis of PF to ERK**

**
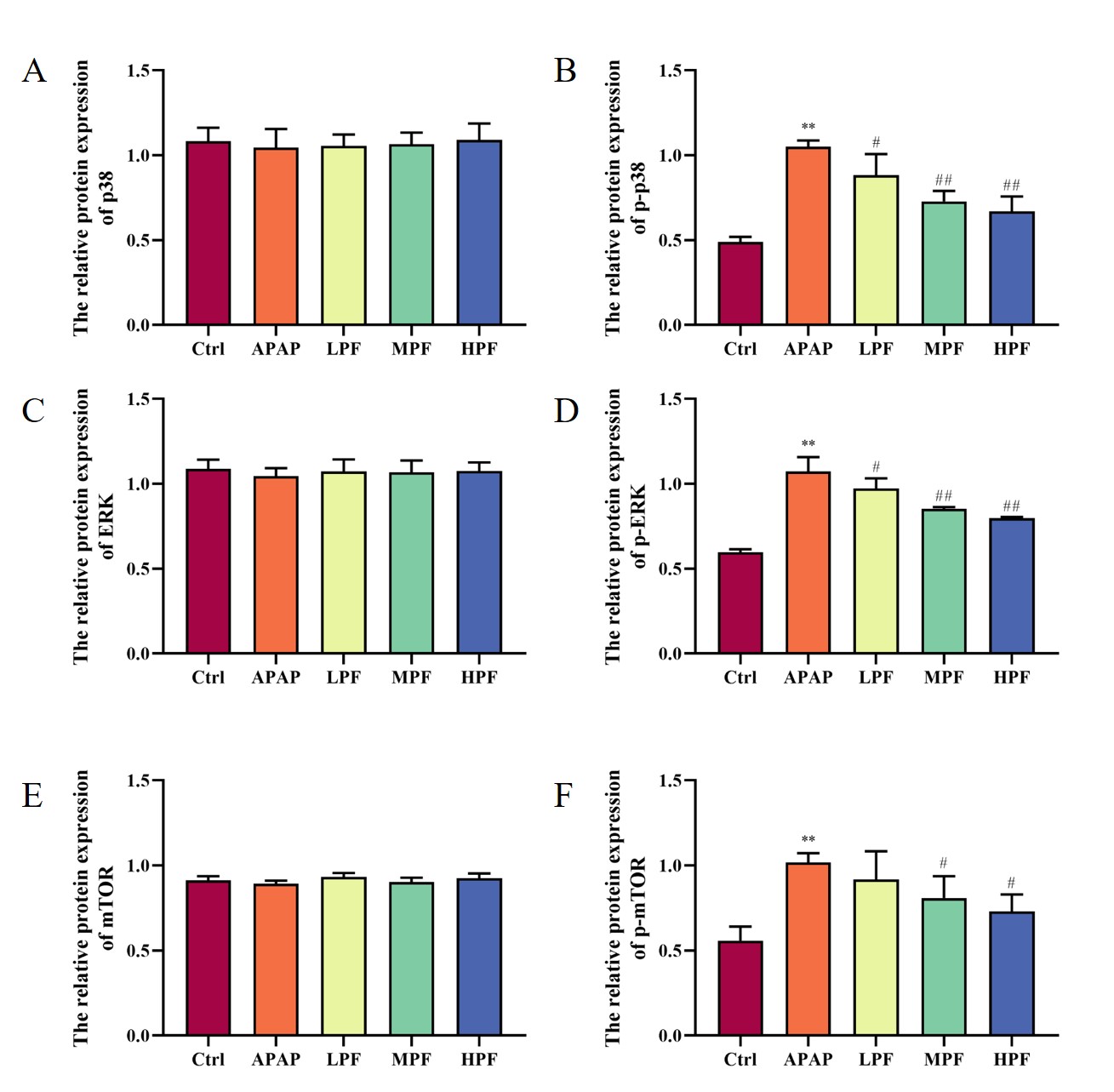
**

**Figure S9 The relative protein expressions of MAPK/mTOR signal**

**(A) the relative protein expression of p38 (B)the relative protein expression of p-p38 (C) the relative protein expression of ERK (D) the relative protein expression of p-ERK (E) the relative protein expression of mTOR (F) the relative protein expression of p-mTOR. Data are presented as mean ± SD in each three samples in group. ^*^*p* < 0.05 and ^**^*p* < 0.01, vs. control; ^#^*p* < 0.05 and ^##^*p* < 0.01, vs. APAP**

**Table S1 Details of chemicals and reagents**

| **Chemicals or reagents** | **Catalogue** | **Manufacturer** |
| --- | --- | --- |
| Acetaminophen | CHB180228 | Chengdu Chroma-Biotechnology Company |
| Paeoniflorin | CHB190124 | Chengdu Chroma-Biotechnology Company |
| Silibinin | 050603005 | Tianjin Tasly Sants Pharmaceutical Co., Ltd |
| Alanine transaminase | C009-2-1 | Nanjing Jiancheng Bioengineering Institute |
| Alkaline phosphatase | A059-2-2 | Nanjing Jiancheng Bioengineering Institute |
| Total bilirubin | C019-1-1 | Nanjing Jiancheng Bioengineering Institute |
| Aspartate transaminase | C010-2-1 | Nanjing Jiancheng Bioengineering Institute |
| γ-glutamyl transpeptidase | C017-2-1 | Nanjing Jiancheng Bioengineering Institute |
| Glutathione | A006-2-1 | Nanjing Jiancheng Bioengineering Institute |
| Superoxide dismutase | A001-3-2 | Nanjing Jiancheng Bioengineering Institute |
| Malondialdehyde | A003-1-2 | Nanjing Jiancheng Bioengineering Institute |

Table S2 Details of experimental grouping and dosage

| **Group** | **Dosage (mg·kg^-1^)** | **Model dosage (mg·kg^-1^)** | **Number** |
| --- | --- | --- | --- |
| Ctrl | — | — | 8 |
| APAP | — | 250 | 8 |
| Sli | 70 |  | 8 |
| LPF | 50 |  | 8 |
| MPF | 100 |  | 8 |
| HPF | 200 |  | 8 |

**Table S3 Details of antibodies used in this study**

| **Antibodies** | **Host** | **Dilution** | **Catalogue** | **Manufacturer** |
| --- | --- | --- | --- | --- |
| **IHC** | | | | |
| CYP2E1 | Rabbit | 1:100 | 19937-1-AP | Proteintech |
| Caspase-9 | Rabbit | 1:100 | 10380-1-AP | Proteintech |
| Caspase-3 | Rabbit | 1:100 | 19677-1-AP | Proteintech |
| p62/SQSTM1 | Rabbit | 1:100 | 18420-1-AP | Proteintech |
| LC3 | Rabbit | 1:100 | 14600-1-AP | Proteintech |
| **WB** | | | | |
| ERK | Rabbit | 1:1000 | 11257-1-AP | Proteintech |
| p-ERK(Thr202/Tyr204) | Rabbit | 1:2000 | 80031-1-RR | Proteintech |
| p38 | Rabbit | 1:1000 | 14064-1-AP | Proteintech |
| p-p38(Thr180/Tyr182) | Rabbit | 1:1000 | 28796-1-AP | Proteintech |
| mTOR | Mouse | 1:1000 | 66888-1-Ig | Proteintech |
| p-mTOR(Ser448) | Mouse | 1:1000 | 67778-1-Ig | Proteintech |
| ULK1 | Rabbit | 1:500 | 29005-1-AP | Proteintech |
| p-ULK1(Ser757) | Rabbit | 1:1000 | #6888 | CST |
| ATG7 | Rabbit | 1:1000 | #2631 | CST |
| ATG5 | Rabbit | 1:1000 | #2630 | CST |
| LC3 | Rabbit | 1:1000 | #3868 | CST |
| p62/SQSTM1 | Rabbit | 1:10000 | 18420-1-AP | Proteintech |
| Bad | Mouse | 1:3000 | 67830-1-Ig | Proteintech |
| Bcl-2 | Rabbit | 1:500 | 26593-1-AP | Proteintech |
| Bax | Mouse | 1:2000 | 60267-1-Ig | Proteintech |
| Caspase-9 | Rabbit | 1:1000 | ab184786 | Abcam |
| Caspase-3 | Rabbit | 1:500 | 19677-1-AP | Proteintech |
| GAPDH | Rabbit | 1:10000 | SA30-01 | HUABIO |
| β-actin | Mouse | 1:10000 | Ab8226 | Abcam |
| Goat anti-rabbit IgG(H+L), HRP conjugate | | 1:3000 | E-AB-1003 | Elabscience |
| Goat anti-mouse IgG(H+L), HRP conjugate | | 1:3000 | E-AB-1001 | Elabscience |

**Table S4 List of primers used for qRT-PCR**

| **Name of genes** | **Forward Primer sequence (5’-3’)** | **Reverse Primer sequence (5’-3’)** | **Annealing temperature (℃)** |
| --- | --- | --- | --- |
| mTOR | AGCTCTGTTTGTGGCTCTGAAT | GCTCCTTGATTCTCCCAATGC | 58.95 |
| Mapk1 | TCTCGTACATCGGAGAAGGC | AGGTCTGGTGCTCAAAAGGA | 57.7 |
| Mapk14 | GACAGGCTACGTGGCTACC | CCCTGGGGTTCCAACGAGT | 56.92 |
| BECN1 | CCAGCGGGAGTATACTGAGT | ATTGTGCCAAACTGTCCGCT | 56.86 |
| Atg7 | CGCCAAGATCTCCTACTCCAATC | TGGCATTCACTCCGGGAAATATT | 59.76 |
| Atg5 | ATGAAGGCACACCCCTGAAA | GGCCCAAAACTGGTCAAATCAT | 55.75 |
| GAPDH | CAGTGGCAAAGTGGAGATTGTTG | TCGCTCCTGGAAGATGGTGAT | 59.01 |

**Table S5 The binding energy of PF to p38 and ERK (kJ/mol)**

| **Compound** | **Target** | **PDB ID** | **Structure of target** | **Affinity (kJ/ mol)** | **Means** | **SD** |
| --- | --- | --- | --- | --- | --- | --- |
| Paeoniflorin | MAPK1 | 1TVO | 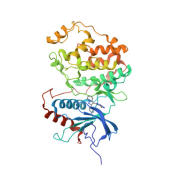 | -22.9338 | -21.6504 | 0.982262 |
|  |  |  |  | -20.54835 |  |  |
|  |  |  |  | -21.46905 |  |  |
|  | MAPK14 | 5ETC | 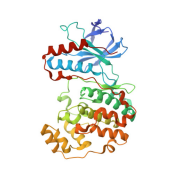 | -31.806 | -32.5035 | 0.711313 |
|  |  |  |  | -33.48 |  |  |
|  |  |  |  | -32.2245 |  |  |
